# HDAC inhibitors in cancer therapy: sensitization mechanisms, combination strategies, and translational challenges

**DOI:** 10.3389/fcell.2026.1951750

**Published:** 2026-09-29

**Authors:** Kun-Yao Liu, Xiang Zhao, Jia-Hui Liu, Mu-Nan Li, Hai-Ying Cui, Zhen Wang, Xin Zhou

**Affiliations:** 1 Cancer Center, The First Hospital of Jilin University, Changchun, China; 2 Laboratory of Organ Regeneration and Transplantation of The Ministry of Education, Changchun, China; 3 China-Singapore Belt and Road Joint Laboratory on Liver Disease Research, The First Hospital of Jilin University, Changchun, China; 4 Cancer Research Institute of Jilin University, The First Hospital of Jilin University, Changchun, China; 5 International Center of Future Science, Jilin University, Changchun, China; 6 Department of Endocrinology and Metabolism, The First Hospital of Jilin University, Changchun, Jilin, China; 7 School of Medicine, Hebei University of Engineering, Handan, China

**Keywords:** combination therapy, histone deacetylase inhibitors, histone deacetylases, sensitizers, synergistic partners

## Abstract

Histone deacetylases (HDACs) are key regulators of chromatin architecture and gene expression, and their dysregulation is closely linked to tumorigenesis, making them attractive targets for cancer therapy. Histone deacetylase inhibitors (HDACis) exert broad antitumor effects by modulating both epigenetic and non-epigenetic pathways. Although HDACis have demonstrated clinical activity in selected hematologic malignancies, their limited efficacy as monotherapy and limited therapeutic benefit in many solid tumors continue to constrain their broader clinical utilization. Accumulating evidence suggests that HDACis may have greater clinical value as treatment sensitizers and synergistic partners in combination regimens. Across chemotherapy, radiotherapy, targeted therapy, endocrine therapy, and immunotherapy, they enhance therapeutic responses through a range of interconnected mechanisms, including chromatin relaxation, impairment of DNA damage repair, induction of apoptosis, suppression of cell cycle progression, inhibition of oncogenic signaling pathways, and remodeling of the immune microenvironment. In this review, we outline the classification and biological functions of HDACs, along with an overview of representative HDACis. We focus on the synergistic and sensitizing roles these agents play across major anticancer modalities and discuss the molecular mechanisms underlying their therapeutic effects. We further discuss key translational barriers, including insufficient isoform selectivity, off-target toxicity, tumor heterogeneity, and inefficient drug delivery. Finally, we highlight emerging strategies—including isoform-selective inhibitors, mechanism-based biomarkers, rationally designed combinations, targeted delivery platforms, and dual- or multi-target agents. Overall, this review provides a comprehensive overview of HDACi-based combination therapies and their therapeutic and translational implications.

## 1 Introduction

Cancer remains a major global public health challenge, characterized by intricate pathogenic mechanisms and profound inter- and intra-tumor heterogeneity. Despite substantial advances in surgery, chemotherapy, radiotherapy, targeted therapy, endocrine therapy, and immunotherapy, persistent clinical challenges—including drug resistance, recurrence, metastasis, and treatment-related toxicity continue to compromise patient outcomes. Consequently, exploring novel strategies to improve therapeutic efficacy has emerged as the forefront of oncology research priorities. While genetic mutations have long been recognized as key drivers of tumorigenesis, mounting evidence now underscores that epigenetic dysregulation plays an equally critical, if not more dynamic, role in cancer initiation, progression, and treatment resistance (Dawson and Kouzarides, 2012). This paradigm shift has opened new avenues for understanding cancer biology and developing innovative therapeutic strategies.

Among the complex network of epigenetic mechanisms driving tumorigenesis, histone acetylation serves as a key regulatory node that balances transcriptional activation and repression (Shvedunova and Akhtar, 2022). This dynamic process is governed by the opposing actions of two enzyme families, with one adding acetyl groups to loosen chromatin and enable gene transcription while the other strips these groups away to compact chromatin and silence gene transcription (Wang et al., 2009). Histone deacetylases (HDACs) are the critical enzymes responsible for this deacetylation process (Taunton et al., 1996). Aberrant activation or overexpression of HDACs is closely linked to various malignancies (Li and Zhu, 2014). Since HDACs exert critical control over chromatin architecture and transcriptional dynamics, they modulate fundamental biological processes in cancer cells such as proliferation, apoptosis, and angiogenesis (Glozak and Seto, 2007; Wang P. et al., 2020). This central role in malignant progression establishes HDACs as highly promising targets for anticancer therapy.

HDACis are a class of epigenetic agents that inhibit HDAC activity to modulate histone acetylation, thereby relaxing condensed compact chromatin structure and altering gene expression. They exert multi-faceted effects on cancer cells and the tumor microenvironment (TME), with malignant cells showing greater sensitivity to HDACis than normal cells (Xu et al., 2007; Bolden et al., 2013). Since the first clinical approval of HDACis, their therapeutic potential has attracted significant attention. Nevertheless, toxicities and limited efficacy in certain tumor types have restricted their clinical application (Shah, 2019; Raucci et al., 2025). Interestingly, extensive studies have demonstrated that HDACis can markedly improve the effectiveness of chemotherapy, radiotherapy, targeted therapy, endocrine therapy, and immunotherapy (Diyabalanage et al., 2013; Liang et al., 2023; Shi et al., 2024). This is achieved by modulating a broad spectrum of cellular processes, ranging from the regulation of DNA repair, cell cycle progression, and apoptosis to the correction of aberrant signaling and the remodeling of the TME (Zhou et al., 2021; Shi et al., 2024). Combining HDACis with other agents not only boosts therapeutic effectiveness but also allows for lower drug doses and reduced toxicity, representing a promising strategy to improve drug sensitivity and expand the spectrum of treatable tumors (Suraweera et al., 2018; Zhou et al., 2021; Ferro et al., 2023). Despite advances in synergistic combination studies and several regimens reaching clinical trials, challenges including incomplete mechanistic insights, suboptimal pharmacokinetics, off-target effects, and insufficient specificity to HDACs continue to limit the clinical translation of combination therapies.

In this review, we systematically outline HDAC classification, biological functions, and representative HDACis, along with the efficacy of HDACis as monotherapy. We prioritize an in-depth exploration of the synergistic mechanisms by which HDACis sensitize tumors to chemotherapy, radiotherapy, targeted therapy, endocrine therapy, and immunotherapy, coupled with a rigorous examination of the underlying molecular pathways. Importantly, solid tumors should not be regarded as a biologically uniform category. The selection and activity of HDACi-based combination strategies may vary substantially across tumor types and molecular subtypes and may be influenced by differences in genomic alterations, oncogenic dependencies, hormone receptor status and hormone dependence, tissue-specific microenvironments, stromal composition, and vascular architecture (Junttila and de Sauvage, 2013; Sanchez-Vega et al., 2018). These factors can shape dominant survival pathways, drug penetration, immune contexture, and resistance mechanisms, thereby influencing the choice and therapeutic activity of specific partner drugs (Dewhirst and Secomb, 2017). Accordingly, HDACi-based combinations should be tailored to the molecular and tissue-specific characteristics of individual tumor types rather than applied uniformly across solid tumors. Beyond discussing clinical translation barriers, such as toxicity, off-target effects, and inefficient drug delivery, we outline several promising future directions, namely, the development of selective HDAC subtype inhibitors, optimized drug delivery systems, and dual- or multi-target agents. By summarizing recent advances and remaining obstacles, this review provides a comprehensive overview of HDACi-based combination strategies in hematologic malignancies and solid tumors.

## 2 Overview of HDACs

### 2.1 HDACs & acetylation

Epigenetic regulation controls gene expression without changing DNA sequences and plays a key role in cell proliferation, differentiation, apoptosis, metabolism, and malignant transformation (Allis and Jenuwein, 2016; Flavahan et al., 2017; Wainwright and Scaffidi, 2017). Histone post-translational modifications, especially the acetylation and deacetylation of lysine residues on histone tails, represent some of the most important and dynamic epigenetic mechanisms. This regulation is controlled by two opposing enzyme families known as histone acetyltransferases (HATs) and HDACs (Chen et al., 2015). HATs add acetyl groups to histones, thus neutralizing the positive charge at the amino terminus and weakening their electrostatic attraction to negatively charged DNA. This relaxed chromatin structure enables transcription factors to access DNA and ultimately promotes gene transcription. Conversely, HDACs remove acetyl groups from histones, increasing their affinity for DNA, triggering chromatin condensation into a more compact structure, and consequently repressing gene transcription. In normal cells, HDACs and HATs coordinate to maintain a balanced level of histone acetylation, ensuring proper gene transcription. In cancer cells, aberrant activation of HATs results in excessive histone relaxing and overexpression of proto-oncogenes; conversely, aberrant activation of HDACs leads to excessive histone condensation, thereby silencing tumor suppressor gene expression. As such, targeting histone acetylation has emerged as a key therapeutic strategy in cancer epigenetic therapy (Figure 1) (Bolden et al., 2006).

**FIGURE 1:**
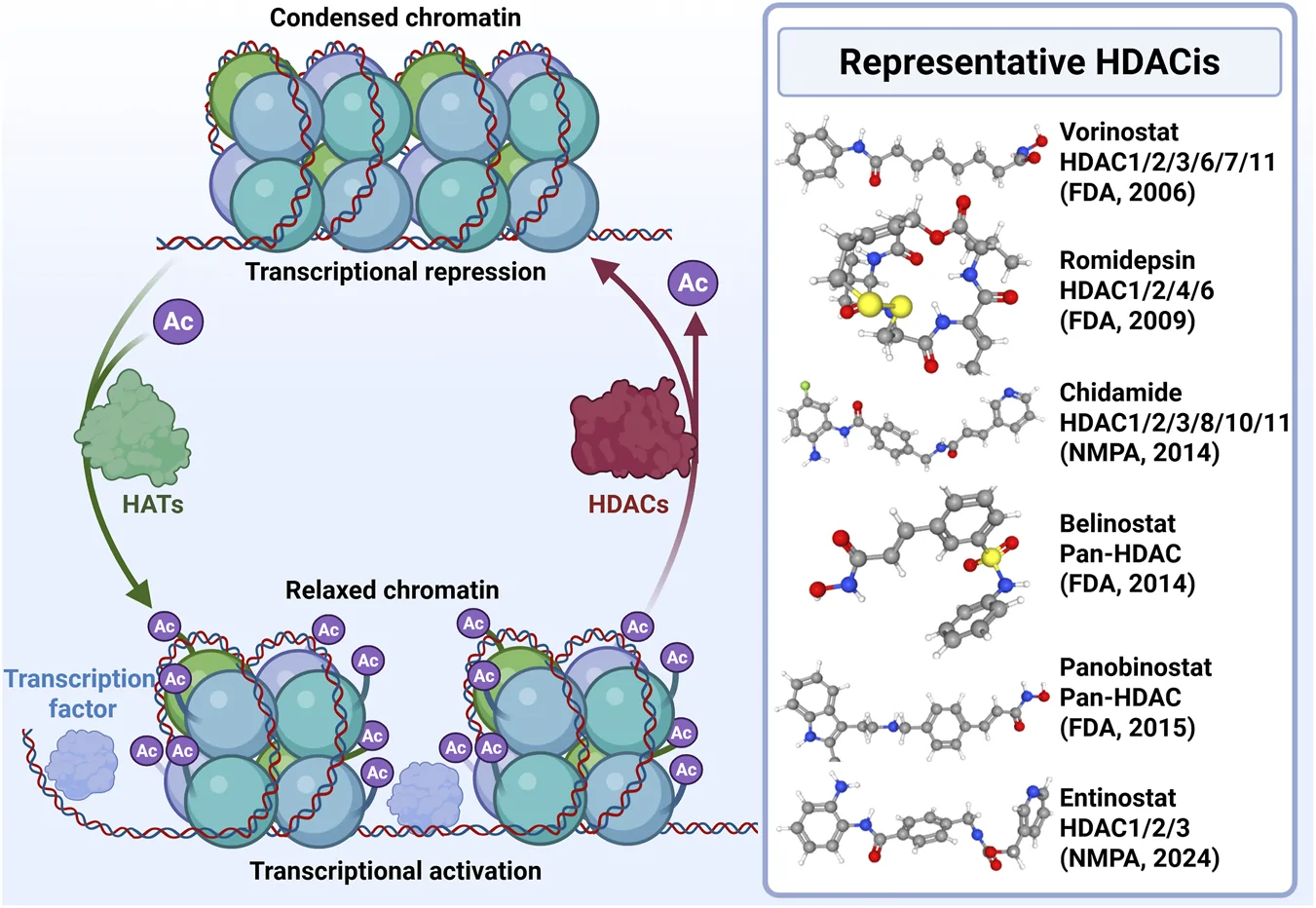
Dynamic regulation of gene transcription by HATs and HDACs via histone acetylation and deacetylation. HAT‐mediated acetylation facilitates transcription factor access to chromatin to drive transcription, while HDAC‐mediated deacetylation represses gene expression. In cancer, aberrant HDAC activity promotes tumorigenesis by silencing tumor suppressor genes. HDACis restore histone acetylation, thereby reactivating tumor suppressor genes and inhibiting tumor growth. The right panel lists representative HDAC inhibitors. Created with BioRender.com and PubChem.The molecular representations were obtained from PubChem. PubChem CIDs: Vorinostat (5311), Romidepsin (5352062), Chidamide (9800555), Belinostat (6918638), Panobinostat (6918837), and Entinostat (4261).

Notably, while HDACs serve as key eraser enzymes for acylation modifications, their catalytic functions extend beyond traditional histone deacetylation. Recent studies have demonstrated that HDACs also remove lactylation (Liu X. et al., 2022; Moreno-Yruela et al., 2022; Xu et al., 2024), crotonylation (Wei et al., 2017; Ntorla and Burgoyne, 2021), succinylation (Li et al., 2023), and other short-chain acyl modifications on histones, thereby participating in broader chromatin-regulatory networks.

Furthermore, the substrate spectrum of HDACs extends beyond histones and encompasses a diverse array of non-histone proteins, including transcription factors, metabolic enzymes, and signaling proteins (Li and Seto, 2016). Among these substrates, transcription factors are of particular importance. For example, HDAC1 is required for the recruitment of GATA-1 to chromatin and for GATA-1-mediated transcriptional regulation. GATA-1 deacetylation and its interaction with HDAC1 modulate its chromatin binding and transcriptional activity, thereby contributing to erythroid and megakaryocytic lineage commitment and differentiation (Yan et al., 2017; Yan et al., 2021).

Similarly, acetylation of estrogen receptor alpha (ERα) regulates its transcriptional activity, protein stability, and hormone sensitivity. The ligand-binding domain of ERα is associated with deacetylase activity and interacts with HDAC1 and HDAC3. Knockdown of either HDAC1 or HDAC3 enhances ERα-mediated transcriptional activity. Moreover, the HDAC inhibitor trichostatin A (TSA) enhances ERα acetylation and protein stability by inducing p300 expression (Kim et al., 2010; Sharma et al., 2017). These mechanisms may be particularly relevant to hormone-sensitive malignancies, including ER-positive breast cancer, in which ERα activity is closely linked to tumor-cell proliferation and therapeutic response.

The functional consequences of HDAC-mediated regulation of non-histone proteins are further illustrated by p53. p53 acetylation induced by HDAC inhibition can modulate p53-dependent cellular stress responses and contribute to its transcription-independent proapoptotic functions, including BAX activation, promotion of reactive oxygen species production, and induction of apoptosis (Yamaguchi et al., 2009; Mrakovcic et al., 2019). These findings further link HDAC inhibition to cellular stress signaling and tumor-cell apoptosis.

Collectively, these examples illustrate that, by regulating non-histone acylation modifications, HDACs participate in cellular processes beyond gene expression, including cell cycle progression, DNA damage repair, metabolism, signal transduction, and autophagy. These findings demonstrate that HDACs are multifunctional enzymes with both epigenetic and non-epigenetic regulatory roles.

### 2.2 Classification and biological functions of HDACs

Based on sequence homology, catalytic mechanism, domain architectures, substrate preference, and subcellular localization, mammalian HDACs are classified into four classes, with class II further divided into classes IIa and IIb (Table 1) (de Ruijter et al., 2003; Gallinari et al., 2007; Seto and Yoshida, 2014).

**TABLE 1:** Classification of HDACs.

Category	Enzyme	Catalytic mechanisms and domain architectures	Intrinsic enzymatic activities and substrate preferences	Subcellular localization and dynamics
Class I	HDAC 1-3&8	Zn^2^^+^‐dependent deacetylases with relatively compact catalytic domains; HDAC1‐3 mainly function within transcriptional corepressor complexes	Robust deacetylation of histone and non-histone proteins; regulation of chromatin compaction and transcriptional repression.	Predominantly localized in the nucleus
Class IIa	HDAC 4, 5, 7, 9	Zn^2^^+^-dependent enzymes with long N‐terminal regulatory domains and C‐terminal catalytic domains	Weak intrinsic deacetylase activity; mainly act as transcriptional coregulators and regulate selected histone and non-histone substrates	Shuttle between the nucleus and cytoplasm; phosphorylation‐dependent nuclear export and import are important for their functions
Class IIb	HDAC 6, 10	Zn^2^^+^‐dependent enzymes with distinct regulatory architectures; HDAC6 contains tandem catalytic domains and a ubiquitin‐binding domain	HDAC6 preferentially targets cytoplasmic proteins; HDAC10 is involved in polyamine deacetylation and autophagy.	HDAC6 mainly cytoplasmic; HDAC10 distributes between the nucleus and cytoplasm
Class III	SIRT 1-7	NAD^+^‐dependent sirtuins with a conserved catalytic core and variable N‐ or C‐terminal targeting/regulatory regions	Catalyze deacetylation and other lysine deacylation reactions; recognize histone, non‐histone, and metabolic substrates	Member-specific localization in the nucleus, cytoplasm, mitochondria, or nucleolus; activity is influenced by NAD^+^ availability and cellular metabolic status
Class IV	HDAC 11	Zn^2^^+^‐dependent enzyme with a catalytic domain related to Class I/II HDACs but distinct substrate and regulatory properties	Exhibits deacetylase and deacylase activities toward selected histone and non‐histone substrates; substrate specificity remains less comprehensively defined	Detected in both the nucleus and cytoplasm; localization and activity are highly dependent on cell type and cellular context

Class I HDACs, including HDAC1, HDAC2, HDAC3, and HDAC8, are homologous to yeast Rpd3 and contain a single Zn^2+^-dependent catalytic domain. They possess relatively high intrinsic deacetylase activity toward histones and nuclear non-histone proteins and are predominantly localized in the nucleus (Mottamal et al., 2015). Class II HDACs are homologous to yeast Hda1 and exhibit more dynamic intracellular localization. Class IIa HDACs, comprising HDAC4, HDAC5, HDAC7, and HDAC9, contain a long N-terminal regulatory interaction domain and a C-terminal HDAC-like catalytic domain. However, substitutions in key catalytic residues substantially reduce their intrinsic deacetylase activity, and they primarily function as signal-responsive transcriptional regulators that shuttle between the nucleus and cytoplasm (Lahm et al., 2007). Class IIb HDACs, including HDAC6 and HDAC10, contain two HDAC-related domains but differ in their catalytic properties and substrate specificities. HDAC6 is predominantly cytoplasmic and preferentially deacetylates non-histone substrates such as α-tubulin and HSP90, whereas HDAC10 also exhibits polyamine deacetylase activity.

Class III HDACs, known as sirtuins (SIRT1-SIRT7), are homologous to yeast Sir2 and utilize NAD^+^ rather than Zn^2+^ as a cosubstrate. Their conserved catalytic core consists primarily of a Rossmann-like NAD^+^-binding domain and a structural zinc-binding domain (Sanders et al., 2010). In addition to deacetylation, several sirtuins catalyze other deacylation reactions, including desuccinylation and demalonylation. Depending on the individual member, sirtuins are localized in the nucleus, cytoplasm, or mitochondria. Class IV contains only HDAC11, which possesses a distinct Zn^2+^-dependent catalytic domain and exhibits unique substrate preferences and biological functions (Núñez-Álvarez and Suelves, 2022). For classes I, II, and IV, Zn^2+^-dependent hydrolysis of the acetyl-lysine amide bond is the basic catalytic mechanism, whereas sirtuins couple deacetylation to NAD^+^ cleavage and nicotinamide release. The major differences among the HDAC classes are summarized in Table 1.

Through both epigenetic and non-epigenetic mechanisms, HDACs regulate a broad spectrum of cellular processes, including cell cycle progression, proliferation, differentiation, apoptosis, DNA damage repair, angiogenesis, metabolism, autophagy, and immune responses (Gallinari et al., 2007; Bahl et al., 2021; Chen et al., 2021). The expression patterns, and biological functions of HDAC isoforms differ across family members. Class I HDACs commonly function within multiprotein complexes and broadly contribute to key physiological processes across human tissues, including chromatin assembly and the maintenance of genomic stability. By modulating programs that govern cell proliferation and survival, aberrant expression of Class I HDACs are associated with tumor progression and poor prognosis (Luo and Li, 2020; Huang et al., 2024). Class II HDACs mainly participate in muscle development and differentiation. They are also involved in a range of biological processes, such as cell migration, immune signaling, and lipid metabolism. Dysregulated expression of Class II HDACs is closely associated with tumor initiation, progression, and metastasis (Malhotra et al., 2019; Travers et al., 2020; Ma et al., 2021; Pulya et al., 2021; Zhou et al., 2022; Martin et al., 2023; Zeleke et al., 2023). Class III HDACs are widely expressed in human tissues, and they regulate key cellular processes, such as inflammation, metabolism, oxidative stress, and apoptosis. They are promising therapeutic targets for diverse disorders, including aging, cancer, cardiovascular, respiratory diseases, and neuropsychiatric conditions (Wu Q. J. et al., 2022; Bonomi et al., 2024). HDAC11, the only class IV member, remains less well characterized, but evidence suggests that it is involved in immune regulation and promotes tumor progression (Wu H. et al., 2022; Liu et al., 2023). Overall, the HDAC family exhibits functional heterogeneity and is dysregulated across multiple malignancies. These features highlight the potential limitations of broad-spectrum HDAC inhibition and support the development of isoform-selective HDAC inhibitors as a strategy for achieving more tumor-specific therapeutic effects. Nevertheless, the therapeutic efficacy of targeting HDACs requires further experimental validation.

## 3 HDACis and anticancer therapy

### 3.1 Classification of HDACis

Members of the HDAC family are frequently overexpressed in various malignant tumors. This aberrant overexpression is closely associated with aggressive cancer behaviors, including uncontrolled proliferation, enhanced cell survival, invasion, and metastasis. As a result, the development of inhibitors targeting HDAC family members has emerged as a major focus in epigenetic cancer research.

HDACis can be categorized based on their chemical structures (Qian et al., 2006; Simonini et al., 2006; Marks, 2007; Atadja, 2009): (1) Hydroxamic acids: These compounds contain a hydroxamic acid moiety (-CONHOH) that chelates zinc ions at the catalytic site. This is the most well-established class, with representative agents including vorinostat (SAHA), panobinostat (LBH589), and belinostat (PXD101). (2) Benzamides: These compounds utilize a benzamide moiety (-CONH_2_) as the zinc-binding group. This class, exemplified by entinostat (MS-275), primarily selectively inhibits Class I HDACs. (3) Cyclic tetrapeptides/cyclic peptides: These compounds feature a cyclic tetrapeptide backbone and employ a thiol group (-SH) for chelation. Romidepsin (FK228) is a representative example. These agents are predominantly selective for Class I HDACs but are structurally complex and challenging to synthesize. (4) Short-chain fatty acids: These compounds possess a carboxyl group (-COOH) and relatively short carbon chains. Examples include valproic acid (VPA) and sodium butyrate. This class exhibits broad-spectrum HDAC inhibition. (5) Other structural classes: Compounds in this category exhibit diverse molecular scaffolds, each with unique mechanisms of action and selectivity profiles, offering distinct pharmacological advantages. To date, several representative HDAC inhibitors have entered clinical development and have been evaluated, particularly in hematologic malignancies (Figure 1). As research advances, the clinical application of HDACis is expected to expand, providing new therapeutic opportunities for a broader range of cancer patients.

### 3.2 Efficacy of HDACis as monotherapy

A series of studies has consistently demonstrated the therapeutic potential of HDACis in the treatment of hematologic malignancies (Bertino and Otterson, 2011; Rashidi and Cashen, 2015; Xu et al., 2015). Their antitumor effects are thought to be attributed to their ability to remodel chromatin architecture, normalize the expression of dysregulated genes, and inhibit the repair of damaged DNA (Valdez et al., 2022). In recent years, while HDACis have yet to achieve the same level of efficacy in solid tumors as observed in hematologic malignancies (Feng et al., 2014), a growing number of studies have begun to uncover their therapeutic potential against certain solid tumors. For example, romidepsin activated both the JNK/c-Jun/caspase-3 pathway and the ERK/CDC25C/CDC2/Cyclin B pathway, thereby inducing apoptosis in hepatocellular carcinoma cells (El Omari et al., 2023); The HDACi JSL-1 exhibited significant inhibitory effects against uveal melanoma by upregulating the expression of the pro-apoptotic BH3-only protein BIM to induce apoptosis, reducing MMP-2 expression to inhibit migration and invasion, and simultaneously blocking the canonical Wnt/β-catenin pathway to impair tumor cell self-renewal capacity (Wang et al., 2017); the HDAC1 inhibitor HR488B exerted antitumor activity by targeting the E2F1/RB/HDAC1 signaling axis, effectively inhibiting colorectal cancer cell proliferation, triggering cell cycle arrest and apoptosis (Duan et al., 2023).

### 3.3 Limitations of HDACis as monotherapy

However, the clinical efficacy of HDACis as monotherapy is often constrained by toxicity, partly owing to the poor isoform selectivity of existing agents. Although only specific HDAC isoforms may contribute to tumor progression, most HDACis broadly inhibit multiple family members in both tumor and normal tissues. Such off-target inhibition, together with altered acetylation of non-histone proteins, may disrupt normal physiological signaling, leading to adverse events such as arrhythmias and dysglycemia (Subramanian et al., 2010; Shah, 2019; Raouf and Moreno-Yruela, 2024; Abdallah, 2026).

An adverse drug reaction (ADR) analysis of several HDACis linked panobinostat to gastrointestinal toxicities, whereas thrombocytopenia was commonly reported with vorinostat, panobinostat, and romidepsin (Begum et al., 2025). Similarly, a real-world pharmacovigilance study based on the FDA Adverse Event Reporting System (FAERS) identified associations between HDACi treatment and serious toxicities including atrial fibrillation, heart failure, respiratory failure, hepatic dysfunction, and acute kidney injury (Li W. et al., 2024). Collectively, these findings suggest that HDACi-related toxicities are diverse and may affect multiple organ systems.

Among these adverse effects, cardiotoxicity represents one of the most clinically significant barriers to the use of HDACis. Romidepsin was the first HDACi to demonstrate clear arrhythmogenic potential in clinical studies (To et al., 2025). In a phase II trial evaluating romidepsin in patients with neuroendocrine tumors, the study was terminated early because of serious cardiac adverse events, including ventricular tachycardia in two patients and sudden cardiac death in one patient (Shah et al., 2006). Likewise, in a phase I trial of intravenous LBH589 in patients with refractory hematologic malignancies, dose-limiting QTcF prolongation was the principal factor restricting treatment tolerability (Giles et al., 2006). This finding is of particular clinical concern because QT prolongation, a surrogate marker of delayed ventricular repolarization, is closely associated with an increased risk of cardiac arrhythmias and may predispose patients to torsades de pointes, ventricular fibrillation, and sudden death (Polak et al., 2009).

In addition to toxicity, suboptimal pharmacokinetic (PK) properties constitute another major barrier to the efficacy of HDACis as monotherapy (McClure et al., 2018; Karagiannis and Rampias, 2021). In non-human primates, Kim et al. used ^11^C-labeled PET imaging to characterize the whole-body distribution of several HDACi-related compounds, and found that butyric acid was rapidly metabolized, whereas VPA and 4-phenylbutyric acid exhibited limited central nervous system (CNS) penetration and minimal brain uptake (Kim et al., 2013). Importantly, these limitations are not confined to short-chain fatty acid derivatives. Hydroxamic acid-based HDACis, despite their potent zinc-chelating capacity and strong *in vitro* activity, frequently exhibit poor plasma stability and rapid clearance, thereby reducing their *in vivo* efficacy (Hermant et al., 2017; Begum et al., 2025). More generally, many HDACis are characterized by short half-lives, rapid systemic clearance, extensive plasma protein binding, and limited penetration across the blood–brain barrier and into the cerebrospinal fluid (BBB/CSF), owing to high molecular weight, elevated polar surface area, and P-glycoprotein-mediated efflux (Begum et al., 2025). These PK liabilities can necessitate higher or more frequent dosing to maintain adequate target engagement, thereby further exacerbating toxicity and limiting the feasibility of HDACis as single-agent therapies.

Moreover, the efficacy of HDACi monotherapy is limited by the development of drug resistance (Fantin and Richon, 2007; Robey et al., 2011; Hao et al., 2024). In breast cancer, vorinostat promotes LIFR expression through BRD4 recruitment, thereby activating the JAK1-STAT3 pathway (Zeng et al., 2016). In renal cell carcinoma, chronic exposure to VPA induces HDACi resistance accompanied by Akt reactivation (Juengel et al., 2013). In pan-HDACi-resistant leukemia cells, vorinostat induces ROS production through NADPH oxidase, resulting in the nuclear translocation of nuclear factor erythroid 2-related factor 2 (NRF2) and the subsequent induction of antioxidant genes as a protective response (Hu et al., 2010). In addition, truncating mutations in *HDAC2* can abolish HDAC2 protein expression or enzymatic activity, thereby confer resistance to HDACis (Ropero et al., 2006). The tumor microenvironment (TME) may also contribute to HDACi resistance. Specifically, HDACi-induced CD47 upregulation suppresses macrophage phagocytosis and promotes them pro-tumor polarization, whereas CD47 blockade restores sensitivity to HDACis (Xu et al., 2025).

Although HDACis are associated with dose-limiting toxicity, off-target effects, inadequate pharmacokinetics and the development of drug resistance, their intrinsic ability to modulate chromatin architecture, which induces chromatin relaxation, upregulates the transcription of pro-apoptotic proteins, inhibits DNA damage repair, and remodels the TME, renders them highly suitable for combination therapy (Figure 2). These multifaceted mechanisms enable HDACis to enhance therapeutic efficacy and generate synergistic effects when used in conjunction with other anticancer approaches. For tumors that are refractory to first-line treatment or have developed resistance following prolonged therapy, integrating HDACis into combination strategies represents a rational and promising therapeutic approach.

**FIGURE 2:**
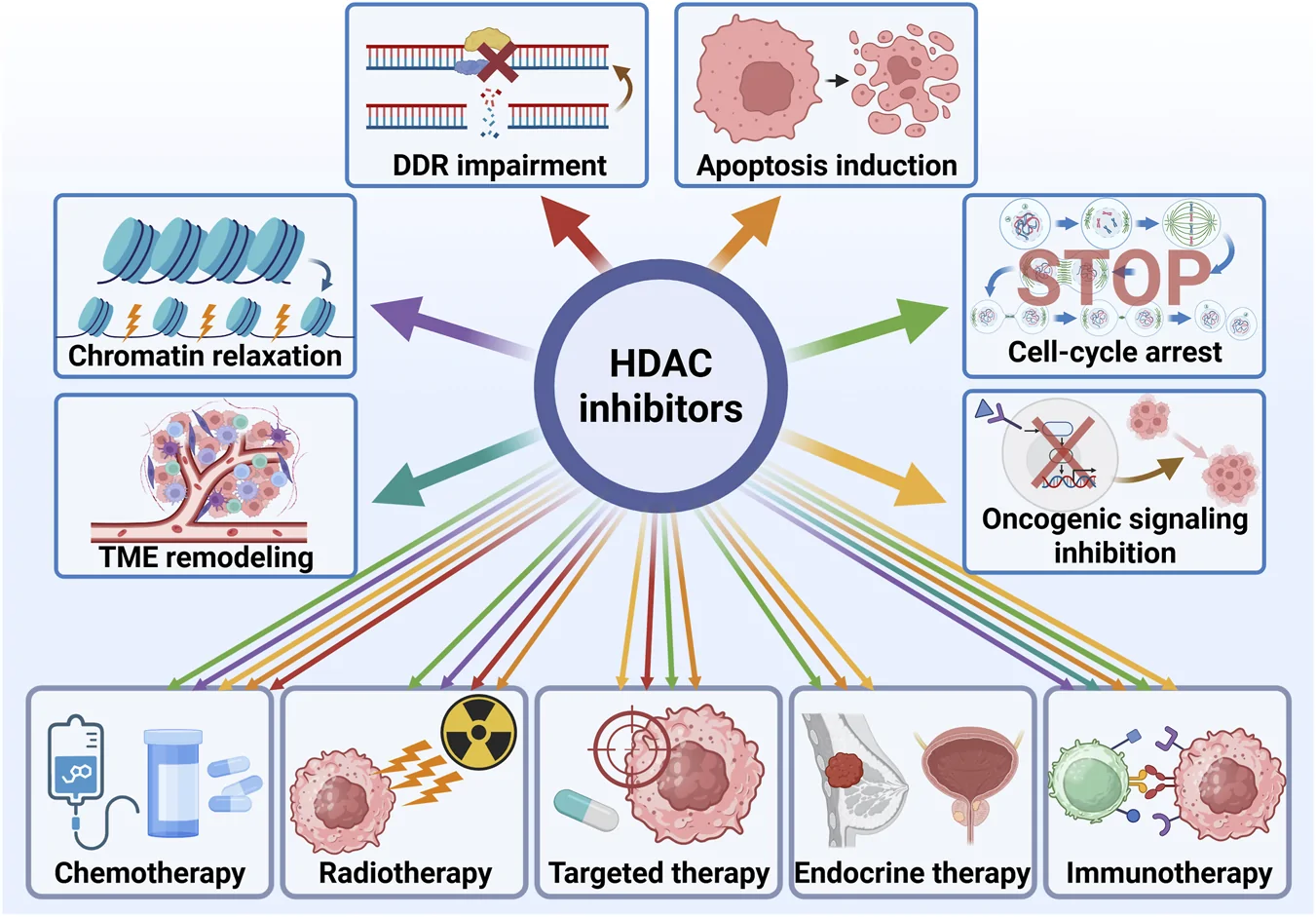
Global mechanistic framework of HDACis-mediated therapeutic sensitization in cancer. HDACis enhance therapeutic responses through interconnected mechanisms, including chromatin relaxation, impairment of DNA damage repair, induction of apoptosis, suppression of cell cycle progression, inhibition of oncogenic signaling pathways, and remodeling of the immune microenvironment. These convergent mechanisms enhance the response to chemotherapy, radiotherapy, targeted therapy, endocrine therapy, and immunotherapy, supporting the rationale for HDACis-based combination treatment strategies. Arrows of the same color link mechanisms with the corresponding therapeutic modalities sensitized by HDACis. Created with BioRender.com.

## 4 Targeting HDACs for sensitization to chemotherapy

As key epigenetic regulators, HDACs enhance the cytotoxicity of chemotherapeutic agents through diverse mechanisms, such as remodeling chromatin structure, promoting apoptosis, and suppressing DNA repair (Figure 3).

**FIGURE 3:**
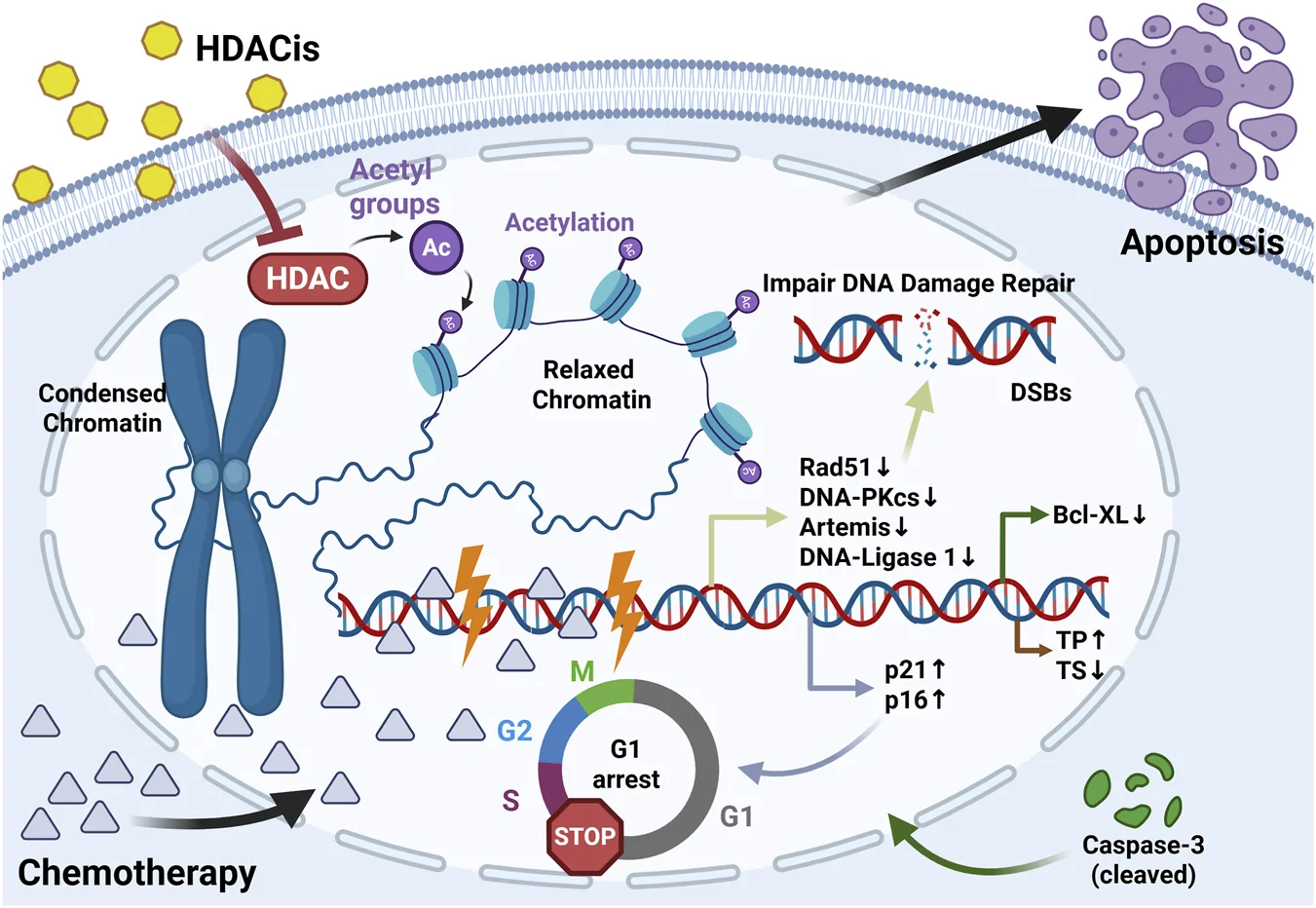
Targeting HDACs to sensitize tumors to Chemotherapy. HDACis enhance chemotherapeutic efficacy by remodeling chromatin, amplifying apoptosis-mediated cytotoxicity, and impairing DNA damage repair processes. Created with BioRender.com.

### 4.1 Chromatin remodeling enhances the accessibility of chemotherapeutics for DNA

Chemotherapeutics often induce cytotoxicity through the formation of DNA adducts, and the compactness of chromatin structure critically influences the binding of these agents to DNA (Jamieson and Lippard, 1999; Wang and Lippard, 2005; Jung and Lippard, 2007). HDACis promote histone hyperacetylation, which disrupts condensed chromatin architecture and enhances DNA accessibility (Zhou et al., 2025). This conformational change increases the binding of chemotherapeutic agents, thereby potentiating DNA damage (Kim et al., 2003). HDACis such as trichostatin A (TSA) and vorinostat were reported to sensitize tumor cells to cisplatin, doxorubicin, and etoposide, particularly when HDAC inhibition preceded chemotherapy (Kim et al., 2003). In gastrointestinal stromal tumor cells, low-dose TSA significantly potentiated cisplatin cytotoxicity by increasing intracellular platinum accumulation and cisplatin-DNA adduct formation (Zhou et al., 2024). Similarly, vorinostat was shown not only to enhance cisplatin-DNA adduct formation but also to alter the distribution of platinum-induced lesions, thereby increasing damage at selected genomic loci (Zacharioudakis et al., 2017). Importantly, this chemosensitizing effect is largely confined to DNA-damaging agents. For instance, VPA enhanced the efficacy of cisplatin and etoposide, but not vincristine, whose antitumor activity was not primarily mediated by direct DNA damage (Groh et al., 2015; Kothari et al., 2016).

Beyond conventional drug combinations, this strategy was also incorporated into nanotherapeutic platforms. In osteosarcoma, carrier-free nano-epidrugs (siMBD-R NPs) co-delivering doxorubicin, siMETTL1, belinostat, and DSPE-PEG2000-cRGD were designed to exploit belinostat-mediated chromatin relaxation, thereby facilitating DNA damage accumulation and enhancing therapeutic efficacy (Qi et al., 2026).

### 4.2 Modulation of apoptotic pathways amplifies cytotoxicity of chemotherapy

HDACis enhance chemotherapy-induced apoptosis in tumor cells by modulating the expression of key apoptotic regulators. For example, entinostat, a class I HDAC inhibitor, induced apoptosis in B-cell lymphoma cells by downregulating the anti-apoptotic protein Bcl-xL and promoting p21-mediated cell cycle arrest. By modulating these apoptotic pathways, it enhanced the cytotoxicity of chemotherapeutic agents such as cytarabine and bortezomib (Frys et al., 2015). In melanoma M14 cells, VPA induced cell cycle arrest and apoptosis through the upregulation of p16, p21, thereby markedly increasing cellular sensitivity to chemotherapeutic agents such as cisplatin and etoposide (Valentini et al., 2007). In colorectal cancer, vorinostat enhanced the efficacy of capecitabine efficacy by upregulating thymidine phosphorylase (TP) and downregulating thymidylate synthase (TS). This combination synergistically inhibited tumor growth and increased apoptosis (Di Gennaro et al., 2010). In diffuse large B-cell lymphoma models, HDACis combined with decitabine synergistically suppressed tumor cell growth and enhanced apoptosis. This time-dependent effect was associated with caspase-3 activation and increased histone acetylation, and was also confirmed in patient-derived cells (Kalac et al., 2011). Similarly, in breast and ovarian cancer cells, a triple combination comprising HDACi (panobinostat or vorinostat), decitabine, and PARP inhibitor markedly elevated the Annexin V-positive rate (42%–59%) and promoted caspase-3 cleavage and activation, thereby potentiating the apoptotic response and amplifying chemotherapy-induced cytotoxicity (Valdez et al., 2024).

### 4.3 Modulation of DNA repair to overcome chemotherapy resistance

The robust DNA repair capacity of tumor cells represents a critical determinant of chemotherapy resistance. HDACis can impair DNA damage repair by modulating the expression of key proteins in DNA repair pathways, thereby enhancing chemosensitivity. For instance, the HDACi PCI-24781 markedly potentiated the cytotoxicity of doxorubicin and cisplatin against soft tissue sarcoma (STS) cells by downregulating RAD51, a pivotal component of DNA homologous recombination repair complexes, thereby impairing the DNA repair capability (Lopez et al., 2009). In addition, a triple-agent combination consisting of HDACi (panobinostat or vorinostat), PARP inhibitor (talazoparib or olaparib), and decitabine induced more pronounced DNA damage in breast and ovarian cancer cells. This enhanced damage was attributed to the downregulation of key DNA damage response proteins, including the non-homologous end-joining factors DNA-PKcs, Artemis, and DNA ligase I, following the treatment with the triple-agent combination (Valdez et al., 2024).

### 4.4 Enhancing the efficacy of chemotherapy via other mechanisms

HDACis facilitate chemosensitization through diverse mechanisms, including the regulation of non-histone protein acetylation and crosstalk with other epigenetic modulators, such as histone lactylation and DNA methylation. In acute myeloid leukemia (AML), HDAC3 promoted chemoresistance by increasing its expression following chemotherapy exposure, which enhanced HDAC3-AKT binding, decreased AKT acetylation, and increased AKT phosphorylation. Accordingly, inhibition or knockdown of *HDAC3* markedly enhanced chemosensitivity and delayed disease progression *in vivo* (Long et al., 2017). In another example, entinostat overcame cisplatin resistance in bladder cancer by promoting H3K18la-mediated DHRS2 expression and nuclear translocation, thereby suppressing the AKR1C3-androgen axis. Of note, high DHRS2 expression and low AKR1C3 expression were correlated with an improved response to neoadjuvant chemotherapy (Xu et al., 2026). Additionally, in AML, HDACi AR-42 enhanced the antileukemic efficacy of decitabine by increasing *miR-29b* levels and decreasing its downstream targets DNMT1, DNMT3A, and DNMT3B, which were key enzymes responsible for DNA methylation (Mims et al., 2013).

### 4.5 Clinical trials of HDACis combined with chemotherapy

Preclinical research has paved the way for the clinical translation of HDACis combined with chemotherapeutic agents, with several such regimens now under clinical investigation. However, the combination therapies evaluated to date exhibit marked disparities in safety, tolerability, and antitumor efficacy. These divergent outcomes provide critical reference data to inform and optimize clinical therapeutic strategies (Table 2).

**TABLE 2:** Representative clinical trials of HDACis in combination with other therapeutic regimens*.

Condition or disease	Phase	Regimen	Primary endpoint	Efficacy and safety	References
Relapsed/refractory multiple myeloma	I; single‐arm trial; n = 20	Panobinostat + Lenalidomide + Bortezomib + Dexamethasone (Pano‐RVd)	Identify MTD of panobinostat in combination with RVd and assess Pano-RVd safety.	This regimen had manageable toxicity (panobinostat MTD, 10 mg); among evaluable patients (n=18), ORR was 44% (90% CI 24–66), ≥VGPR was 17%, and CBR (≥MR) was 61% (90% CI 39–80).	NCT01965353 (Laubach et al., 2021)
Refractory sarcoma	I; single‐arm trial; n = 40	Panobinostat + Epirubicin	Safety/toxicity and phase II recommended dose determination.	This regimen was well tolerated (panobinostat MTD, 50 mg). In the sarcoma dose expansion cohort (n=20), 1 patient achieved PR, 11 had SD >3 months; median PFS 3.4 months (95% CI 2.6–5.2), OS 8.3 months (95% CI 7.1–10.6).	NCT00878904 (Thomas et al., 2016)
Recurrent/metastatic squamous cell carcinomas	II; single‐arm trial; n = 112	Vorinostat + Pembrolizumab	ORR in each tumor cohort during treatment	Evaluable patients (n = 107): the ORR was met in cervical (39%), anal (31%), and vulvar/vaginal (19%) cohorts, but not HNSCC (19%) or penile cancer (18%). Median PFS 4.0 months (95% CI, 2.6–4.3); median OS 11.1 months (95% CI, 9.2–17.4). Grade ≥3 TRAEs occurred in 44.4% of the safety population.	NCT04357873 (Borcoman et al., 2025)
Previously untreated peripheral T‐Cell lymphoma	II; single‐arm trial; n = 29	Romidepsin + Lenalidomide	ORR	The ORR was 65.2% (95% CI, 42.7‐83.6) with CR at 26.1% (95% CI, 10.2‐48.4) for all evaluable patients (n = 23). Grade ≥3 TRAEs mainly included hematologic toxicities, hyponatremia, hypertension, and infections.	NCT02232516 (Ruan et al., 2023)
High-risk stages I and II of ENKTL	II; single‐arm trial; n = 37	Chidamide + Tislelizumab + Pegaspargase + Radiotherapy	CRR and ORR after 6 induction cycles.	Efficacy-evaluable (n=28): median follow‐up, 36.5 months; median PFS/OS, not reached. But treatment completers (n=24): CRR/ORR, 100%. Grade ≥3/4 AEs mainly included elevated ALT/AST (16.2%), neutropenia (8.1%), anemia, thrombocytopenia, and upper gastrointestinal hemorrhage (5.4% each).	NCT04414969 (Li et al., 2024b)
Advanced soft tissue sarcoma	II; single‐arm trial; n = 69	Chidamide + Toripalimab	ORR assessed by RECIST 1.1	Efficacy-evaluable (n=69): ORR 29%; DCR 73.9%; median time to an initial response, 5.0 months (95% CI, 3‐7); median PFS, 7.1 months (95% CI, 4.0‐10.2); median OS not reached. Treatment was well tolerated, with most adverse events being grade 1‐2.	NCT04025931 (Zhang et al., 2025)
Previously untreated AITL	II; single‐arm trial; n = 71	Chidamide + Prednisone + Etoposide + Thalidomide	ORR and CRR after eight cycles	Completed eight CPET cycles (n=51): ORR 90.2%, CRR 54.9%. After a median follow‐up of 11.4 months (95% CI, 9.9–17.0), median PFS was 42.6 months (95% CI, 27.7‐not reached), while median OS was not reached. The most common grade 3/4 AE: neutropenia (n = 22, 32.3%).	NCT03273452 (Wang et al., 2022)
AML	II; single‐arm trial; n = 30	Chidamide + CAG + Venetoclax + Azacitidine (CACAG‐VEN)	ORR after one cycle of induction	All patients completed one CACAG‐VEN cycle. ORR 96.7% (95% CI, 83.3–99.8), CRc rate 93.3% (95% CI, 78.7–98.8). The most common grade 3/4 AE: febrile neutropenia (40.0 %).	NCT05659992 (Yang et al., 2025)
Pancreatic ductal adenocarcinoma	II; single‐arm trial; n = 27	Entinostat + Nivolumab	ORR assessed by RECIST 1.1	Primary PFS endpoint not met; ORR 11% (95% CI, 2.4%‐29.2%); Grade ≥3 TRAEs occurred in 63% of patients.	NCT03250273 (Baretti et al., 2024)
HR+ advanced breast cancer	III; RCT; n = 354	Entinostat + Exemestane vs Placebo + Exemestane	Independent radiographic committee (IRC)‐assessed PFS	Primary IRC‐assessed PFS endpoint met; longer PFS with entinostat + exemestane vs placebo + exemestane (6.32 vs 3.72 months; HR, 0.76 [95% CI, 0.58‐0.98]; P = 0.046). Grade ≥3 AEs were more frequent with entinostat (65.5% vs 19.3%).	NCT03538171 (Xu et al., 2023)
Previously untreated peripheral T‐Cell lymphoma	III; RCT; n = 421	Ro‐CHOP vs CHOP	PFS	Primary PFS endpoint not met; no significant PFS, OS, or response rate benefit with Ro‐CHOP vs CHOP, with increased grade 3‐4 hematologic toxicities. An exploratory TFH lymphoma subgroup: longer PFS with Ro‐CHOP (19.5 vs 10.6 months; HR, 0.703[95% CI, 0.502‐0.985]; P =0.039); this finding should be considered hypothesis‐generating.	NCT01796002 (Camus et al., 2024)

^*^ Because of space limitations, only representative clinical trials with available efficacy, safety, and endpoint information are included. The studies are presented according to their design and level of clinical evidence.

*CHOP*, Cyclophosphamide, Doxorubicin, Vincristine and Prednisone;*CPET*, Chidamide, Prednisone, Etoposide and Thalidomide;*CAG*, cytarabine, aclarubicin, and G-CSF;*AML*, acute myeloid leukemia;*MDS*, myelodysplastic syndrome;*ENKTL*, Extranodal NK/T-Cell Lymphoma;*PTCL-TFH*, Peripheral T-Cell Lymphoma with T Follicular Helper Phenotype;*AITL*, Angioimmunoblastic T-cell Lymphoma;*MTD*, maximum tolerated dose;*ORR*, objective response rate;*CRR*, complete response rate;*CI*, confidence interval;*VGPR*, very good partial response;*CBR*, clinical benefit response;*MR*, minimal response;*PR*, partial response;*SD*, stable disease;*PFS*, progression-free survival;*OS*, overall survival;*TRAE*, treatment-related adverse event;*CR*, complete response;*AE*, adverse event;*DCR*, disease control rate;*CRc*, composite complete remission.

In a phase I study, the HDAC6 inhibitor citarinostat (ACY-241) plus paclitaxel was well tolerated in patients with advanced solid tumors, and 360 mg/day was chosen for phase II expansion due to acceptable safety, increased acetylation, and preliminary efficacy (Gordon et al., 2021). Similarly, chidamide plus paclitaxel and carboplatin was feasible in advanced NSCLC, with 20 mg/day twice weekly being the recommended dose (Hu et al., 2016). Likewise, a phase II trial in advanced non-small cell lung cancer (NSCLC) showed that adding vorinostat to carboplatin and paclitaxel improved the confirmed response rate and was associated with trends toward longer progression-free and overall survival, but grade 4 platelet toxicity occurred more frequently in the vorinostat arm. (Ramalingam et al., 2010). In HR^+^/HER2^−^ breast cancer, chidamide combined with epirubicin, cyclophosphamide, and docetaxel achieved a residual cancer burden 0/I rate of 35.2% (19 of 54, 95% CI, 22.7%–49.4%) with acceptable safety (Xue et al., 2025). Overall, HDACis have the potential to enhance the efficacy of chemotherapy in selected settings, and future studies are warranted to further optimize HDACi-chemotherapy combination strategies.

In summary, HDACis enhance chemosensitivity through chromatin relaxation, promotion of apoptosis, suppression of DNA repair, and modulation of non-histone or epigenetic pathways. In parallel, clinical trials have demonstrated the safety and efficacy of several HDACi-chemotherapy combination strategies.

## 5 Targeting HDACs for sensitization to radiotherapy

Radiotherapy is a cornerstone in the treatment of solid tumors, eradicating cancer cells primarily through ionizing radiation-induced DNA damage; however, the reduced sensitivity of tumor cells to radiation often limits its therapeutic efficacy. Accumulating evidence suggests that HDACi enhance the radiosensitivity of tumor cells via various mechanisms, such as modulating chromatin architecture, disrupting DNA damage repair, and altering cell cycle progression, thus offering a promising strategy to enhance the efficacy of radiation therapy (Figure 4).

**FIGURE 4:**
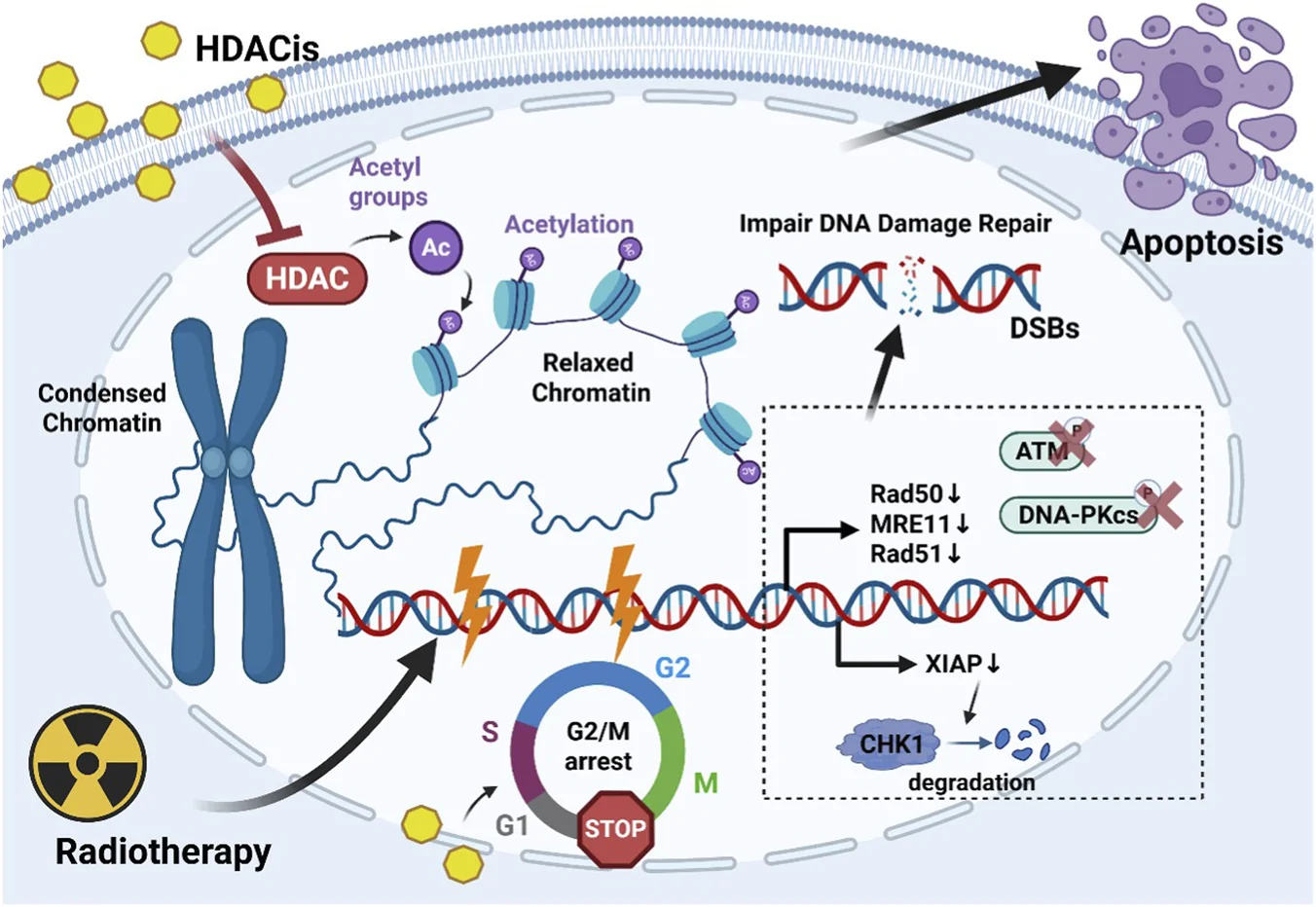
Targeting HDACs to sensitize tumors to Radiotherapy. HDACis enhance radiotherapeutic efficacy by remodeling chromatin, impairing DNA damage repair processes, and inducing cell cycle arrest. Created with BioRender.com.

### 5.1 Chromatin remodeling amplifies radiation-induced DNA damage

The extent of chromatin condensation directly affects the efficacy of radiation-induced DNA damage (Li et al., 2018; Brambilla et al., 2020). By inhibiting HDACs and enhancing histone acetylation, HDACis facilitate the relaxation of highly condensed chromatin. This structural remodeling creates additional targets for radiation-induced DNA damage, thereby amplifying the cytotoxicity of radiotherapy (Zhao et al., 2025).

Acquired radioresistant breast cancer cells exhibited compact heterochromatin and reduced histone acetylation, whereas HDACis reversed these features and restored sensitivity to radiation-induced DNA damage (Sharda et al., 2020). Similarly, increased heterochromatin compaction was associated with radioresistance in prostate stem-like cells, while HDACis treatment relaxed chromatin and enhanced radiation-induced cytotoxicity (Frame et al., 2013). Further studies showed that vorinostat increased histone H4 acetylation and induced a more open chromatin state, thereby facilitating radiation-induced DNA breaks formation in accessible chromatin regions (Singh et al., 2010). In multiple myeloma cells, vorinostat also enhanced radiation-induced γ-H2AX accumulation at low concentrations, further supporting the role of chromatin relaxation in HDACi-mediated radiosensitization (Chen et al., 2012).

This concept has also been translated into nanoparticle-based delivery strategies. In murine models of colorectal and prostate carcinoma, nanoparticle formulations of HDACis retained their chromatin-modulating activity and increased the accessibility of DNA to radiation, thereby enhancing the accumulation of DNA damage (Wang et al., 2015). Likewise, Zhu et al. developed redox-sensitive iodinated polymersomes (RIP) carrying vorinostat as a dual-functional nano-radiosensitizer in breast cancer, which significantly increased tumor DNA damage with tolerable toxicity (Zhu et al., 2021).

### 5.2 Interference with DNA damage repair pathways enhances radiosensitivity

One key mechanism by which HDACis enhance radiotherapy efficacy lies in their ability to selectively impair DNA damage repair in cancer cells (Groselj et al., 2013). Normal cells effectively repair radiation-induced damage, while tumor cells, with inherent repair deficiencies, accumulate damage and die. Using γ-H2AX as a marker of DNA double-strand breaks (DSBs), vorinostat was shown to increase DSB formation in both normal fibroblasts and cancer cells. However, whereas normal cells rapidly resolved γ-H2AX signals, cancer cells exhibited sustained γ-H2AX accumulation due to reduced expression of key DNA repair proteins, including RAD50 and MRE11, thereby enhancing radiosensitivity (Lee et al., 2010). The interaction of HDACs with key DNA damage response factors is also a major mechanism by which HDACs regulate DNA repair pathways. Notably, ATM acts as a critical sensor of radiation-induced DNA damage and coordinates DNA repair. Kim et al. demonstrated that ATM recognizes DNA damage by interacting with HDAC1, with the binding being increased after ionizing radiation in human fibroblast cells (Kim et al., 1999). Subsequently, ATM activates BRCA1, CHK2, and p53, upregulating repair-related genes such as *CDKN1A*, *GADD45A*, and *RRM2B* to induce cell cycle arrest and DNA damage repair (Thurn et al., 2013). In parallel, HDAC inhibition can suppress ATM expression and p53 activation. Selective knockout of *HDAC1/2* significantly reduced the expression of DNA repair-associated genes, including *GADD45A* and *RRM2B*, thereby enhancing cellular sensitivity to DSBs (Thurn et al., 2013). Recently, isoform-specific inhibition of HDAC3 was reported to radiosensitize fusion-positive rhabdomyosarcoma by impairing DNA damage repair following irradiation. HDAC3 depletion or pharmacologic inhibition with MC4448 increased irradiation-induced γ-H2AX accumulation and DSBs, accompanied by suppressed activation of ATM and DNA-PKcs, as well as reduced RAD51 expression (Cassandri et al., 2024). Using yeast as a model, Ortega et al. revealed that the Rpd3L and Hda1 histone deacetylase complex, the yeast counterpart of human HDAC1-containing complexes, promoted the repair of replication-born DSBs by facilitating cohesin loading. This HDAC-dependent cohesion acted specifically without affecting other homology-dependent repair pathways, thus preventing genome instability (Ortega et al., 2019). In glioblastoma multiforme, HDAC6 is highly expressed in glioma stem cells, a key population that drives radioresistance. Yang et al. demonstrated that HDAC6 inhibition impaired DNA damage repair by downregulating XIAP expression to promote CHK1 degradation, while also reducing stem cell characteristics by blocking the SHH/Gli1 pathway. These dual mechanisms collectively enhance glioblastoma stem cell radiosensitivity, suggesting HDAC6 as a potential target for overcoming radiation resistance in solid tumors (Yang et al., 2018).

### 5.3 Cell cycle modulation expands the therapeutic window for radiation treatment

Cellular radiosensitivity varies considerably across the phases of the cell cycle: G2/M-phase cells are highly radiation-sensitive, whereas S-phase cells are relatively resistant due to their robust DNA repair ability (Pawlik and Keyomarsi, 2004; Fan et al., 2020). HDACis can induce G2/M arrest in tumor cells, thereby increasing the proportion of radiosensitive cells and broadening the therapeutic window of radiotherapy. Belinostat, a pan-HDAC inhibitor, induced G2 phase arrest in rhabdomyosarcoma cells to enhance radiosensitivity. It also increased radiation-induced ROS accumulation and impaired ROS detoxification (Marampon et al., 2019). In esophageal cancer cells, TSA further promoted G2/M arrest induced by radiation, and increased apoptosis (Wang et al., 2021). Similarly, in bladder cancer cells, panobinostat increased γ-H2AX foci formation in combination with radiotherapy compared with radiotherapy alone, and further enhanced radiation-induced G2/M arrest (Tsai et al., 2023). In NSCLC cells, TSA significantly reduced cell survival after γ-irradiation and enhanced ionizing radiation-induced G2/M arrest, accompanied by upregulation of p21 (Zhang F. et al., 2009).

### 5.4 Clinical trials of HDACis combined with radiotherapy

Clinical evidence indicates that HDACis have the potential to enhance the efficacy of radiotherapy. In relapsed or refractory neuroblastoma, vorinostat plus MIBG produced a higher response rate than either MIBG alone or MIBG combined with vincristine and irinotecan, with a manageable safety profile. (DuBois et al., 2021). In patients with gastrointestinal cancers, vorinostat showed acceptable safety when combined with short-term pelvic palliative radiotherapy. The phase I study established 300 mg once daily as the maximum tolerated dose, while histone hyperacetylation provided evidence of target engagement (Ree et al., 2010). In patients with head and neck squamous cell carcinoma, vorinostat combined with chemoradiotherapy showed manageable toxicity and established the maximum tolerated dose of vorinostat at 300 mg every other day. (Teknos et al., 2019). In addition, in patients with advanced solid tumors, abexinostat was tolerable when combined with hypofractionated radiotherapy, but its antitumor activity was modest (Deutsch et al., 2017). Overall, these studies indicate that HDACis-radiotherapy combinations are clinically feasible and generally tolerable, with antitumor activity in selected settings; however, the variability in efficacy highlights the need for further studies to optimize patient selection, dosing strategies, and combination regimens.

In conclusion, HDACis markedly enhance tumor cell sensitivity to radiation via multiple mechanisms, including the regulation of chromatin structure, disruption of DNA damage repair pathways, and modulation of the cell cycle progression. Clinical trials suggest that HDACis have the potential to enhance the efficacy of radiotherapy; however, this finding still requires validation in larger clinical cohorts.

## 6 Targeting HDACs for sensitization to targeted therapy

### 6.1 Enhancing sensitivity to RTKs, MAPK, and PI3K-AKT-mTOR inhibitors via HDAC inhibition

Aberrant activation of receptor tyrosine kinases (RTKs) and their downstream signaling cascades, including the PI3K-AKT-mTOR and MAPK pathways, is a key driver of tumorigenesis and tumor progression (He et al., 2021; Ullah et al., 2022; Tomuleasa et al., 2024). Through epigenetic and non-epigenetic mechanisms, HDACis have been shown to enhance the anticancer efficacy of inhibitors targeting these pathways (Figure 5).

**FIGURE 5:**
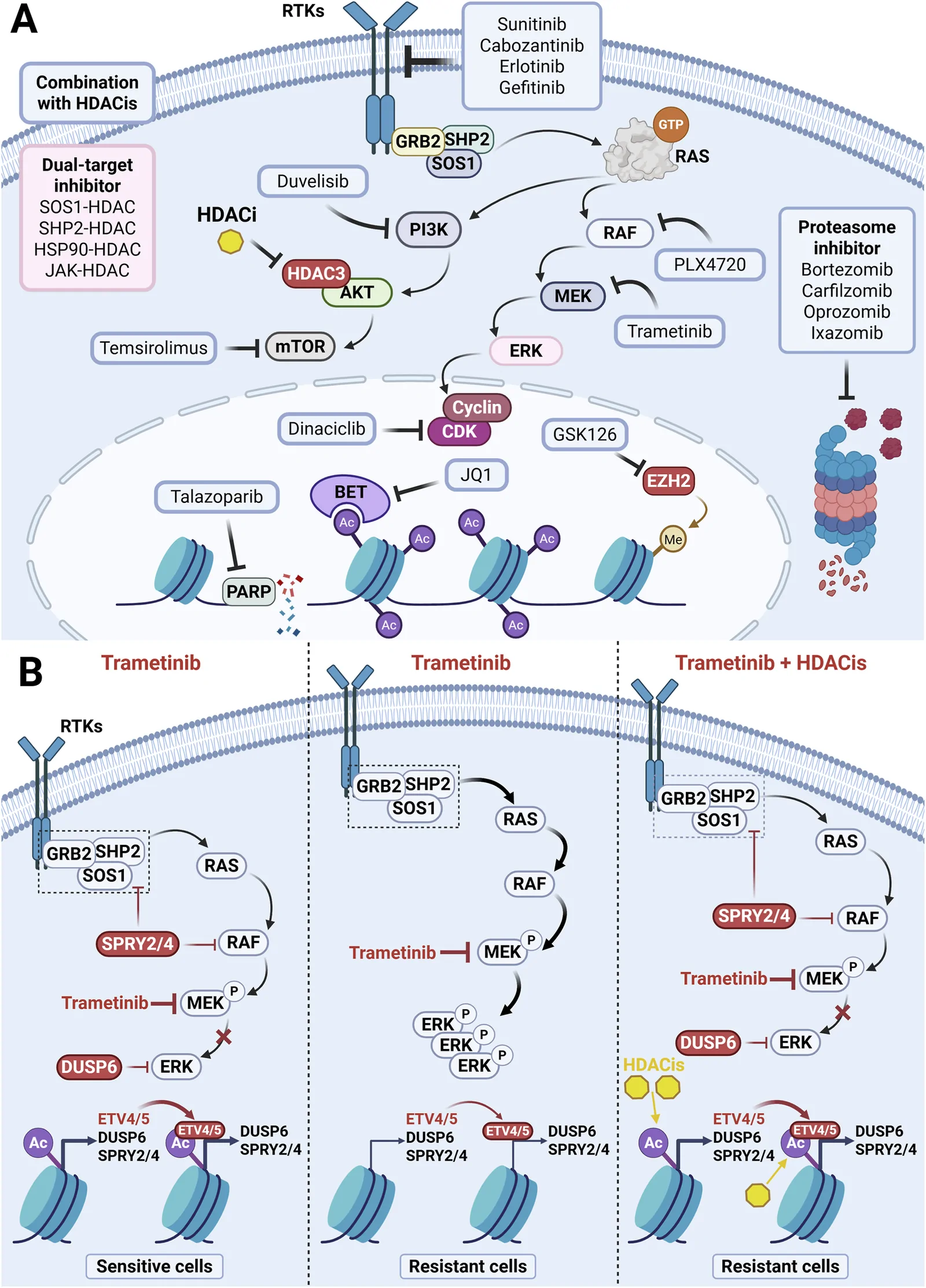
Targeting HDACs to sensitize tumors to Targeted Therapy. **(A)** HDACis enhance the sensitivity of cancer cells to a wide range of targeted therapies, including inhibitors of RTKs, the downstream MAPK and PI3K-AKT-mTOR pathways, the proteasome, CDK4/6, PARP, EZH2, and BET. Furthermore, dual-targeting HDACis which simultaneously inhibit HDACs and key signaling molecules can further improve therapeutic efficacy. **(B)** Representative case of HDACi in combination with targeted therapy: in ovarian cancer, resistance to the MEK inhibitor trametinib is closely linked to enhancer reprogramming. In sensitive cells, trametinib blocks MEK signaling, while H3K27ac-marked enhancers drive the expression of MAPK negative regulators such as DUSP6, ETV4, ETV5, SPRY2, and SPRY4. In resistant cells, these enhancers lose H3K27ac, leading to reduced expression of these negative regulators, loss of MAPK feedback inhibition, and acquisition of drug resistance. HDACis can restore H3K27ac at these enhancers, reactivate negative regulator expression, and resensitize cells to trametinib. Created with BioRender.com.

#### 6.1.1 Targeting HDACs for sensitization to RTK inhibitors

HDACis can enhance the efficacy of RTK inhibitors, particularly those targeting epidermal growth factor receptor (EGFR) (Du et al., 2025). Romidepsin synergistically enhanced the sensitivity of *EGFR* wild-type, mutant, and resistant NSCLC cells to erlotinib and inhibited xenograft tumor growth by inducing apoptosis (Zhang W. et al., 2009). Similarly, in NSCLC, activation of compensatory signaling pathways limited the efficacy of gefitinib and erlotinib; however, vorinostat improved therapeutic efficacy by increasing ROS levels through upregulation of VDAC1 and modulation of the c-Myc-NRF2-KEAP1 axis (Leone et al., 2015). *HDAC6* overexpression in lung adenocarcinoma stabilized EGFR protein, thereby contributing to resistance. Consistently, the selective HDAC6 inhibitor CAY10603 reduced EGFR levels and synergized with gefitinib to promote apoptosis (Wang et al., 2016). In addition to its role in EGFR signaling, HDAC3 was also reported to increase FGFR1 expression via NKX2.1-dependent transcriptional regulation, thereby promoting *KRAS*-mutant tumor progression. Importantly, entinostat suppressed this HDAC3-mediated FGFR1 signaling and restored sensitivity to targeted therapy (Eichner et al., 2023). In addition, HDACis can also enhance the efficacy of multi-targeted RTK inhibitors such as sunitinib and cabozantinib. In clear cell renal cell carcinoma, HDAC8 decreased sensitivity to sunitinib by promoting HIF-2α activity through ETS1 deacetylation, whereas HDAC8-targeting PROTACs restored sunitinib sensitivity by inducing HDAC8 degradation (Qian et al., 2024). In hepatocellular carcinoma, romidepsin reprogrammed lipid metabolism, disrupted mitotic spindle function, and modulated cell cycle- and survival-related pathways, thereby creating a state of vulnerability in which tumor cells became more dependent on RTK signaling for survival and were consequently more susceptible to cabozantinib-induced cytotoxicity (Sequera et al., 2025).

#### 6.1.2 Targeting HDACs for sensitization to PI3K-AKT-mTOR inhibitors

Aberrant activation of the phosphoinositide 3-kinase (PI3K)-AKT serine/threonine kinase (AKT)-mammalian target of rapamycin (mTOR) pathway is a major driver of tumor initiation and progression (He et al., 2021; Glaviano et al., 2023). HDAC inhibition has emerged as a promising strategy to enhance the efficacy of inhibitors targeting this signaling axis. In *TP53*-mutant diffuse large B-cell lymphoma, the combination of HDACis with the PI3Kδ inhibitor duvelisib synergistically inhibited HDAC2 and PI3Kδ, suppressed autophagy, and induced apoptosis via stabilization of cytoplasmic IκBα (Yao et al., 2025). In medulloblastoma, panobinostat synergized with PI3K inhibitors to suppress the growth of *MYC*-driven tumors, at least in part by inducing the expression of the tumor suppressor gene *FOXO1* (Pei et al., 2016). AKT, a downstream effector that plays a well-established role in the development of drug resistance, is also subject to HDAC-mediated regulation (Song et al., 2019). Long et al. demonstrated that HDAC3 promoted chemotherapy resistance by interacting with AKT and deacetylating AKT at Lys20, thereby enhancing AKT phosphorylation. Conversely, HDAC3 inhibition reversed this effect and sensitized leukemia cells to chemotherapy-induced apoptosis (Long et al., 2017). As a key downstream effector of the PI3K-AKT pathway, mTOR is an important therapeutic target in multiple cancers (Mossmann et al., 2018). However, resistance can frequently emerge after prolonged treatment with mTOR inhibitors such as everolimus and temsirolimus. In bladder cancer, VPA inhibited the growth of temsirolimus-resistant cancer cells and induced G0/G1 cell cycle arrest by downregulating *CDK1*, Cyclin B, *CDK2*, Cyclin A, and SKP1/p19 while upregulating p27 (Juengel et al., 2017). In addition, VPA increased histone H3/H4 acetylation and suppressed AKT-mTOR signaling, suggesting that the combination of temsirolimus and VPA may represent a promising therapeutic strategy for bladder cancer (Juengel et al., 2017).

#### 6.1.3 Targeting HDACs for sensitization to MAPK inhibitors

MAPK inhibitors targeting rapidly accelerated fibrosarcoma kinase (RAF), mitogen-activated extracellular signal-regulated kinase kinase (MEK), or extracellular signal-regulated kinase (ERK) are widely used in cancer therapy; however, therapeutic outcomes vary across tumor types and treatment settings, and resistance often emerges (Ullah et al., 2022). HDAC8 was upregulated in melanoma with acquired resistance to BRAF inhibitors and promoted resistance by activating RTKs, leading to aberrant MAPK pathway reactivation and enhanced c-Jun transcriptional activity. Conversely, HDAC8 inhibitors improved the durability of BRAF-targeted therapy (Emmons et al., 2019). The therapeutic efficacy of MEK inhibitors can also be constrained by resistance mechanisms in some cases (Hong et al., 2021; Daley et al., 2023). HDACis can restore drug sensitivity and exert synergistic antitumor effects when combined with MEK inhibition. In pancreatic cancer, MPT0E028 enhanced the antitumor activity of MEK inhibitors in both *KRAS*-mutant and wild-type cells by promoting apoptosis (Chao et al., 2019). In ovarian cancer, trametinib resistance was associated with enhancer reprogramming that suppressed MAPK negative regulators and activated ERK; importantly, HDACis reversed this process, restored these regulators, and synergized with trametinib to inhibit tumor growth (Liu et al., 2021). In *BRAF* ^*V600E*^-mutant microsatellite stable tumors, HDACis and MEK inhibitor synergistically suppressed EGFR-, MAPK-, and PI3K-AKT-related signaling pathways and downregulated the key target HOXC6. In other *BRAF*-mutant colorectal cancer models, HDACis inhibited c-MET/STAT3-mediated c-FLIPL induction and sensitized cells to MEK inhibitor-induced cell death (Carson et al., 2015; Sun et al., 2025). In addition, combined HDAC and MEK/ERK inhibition induced apoptosis in colorectal cancer and suppressed *APC*-driven adenoma formation by increasing BIM and BMF expression (Jenkins et al., 2023).

#### 6.1.4 Dual-action inhibitors targeting HDACs and RTK-Related signaling pathways to achieve superior efficacy

Beyond combination therapy, dual-target inhibitors that simultaneously inhibit HDACs and key nodes in RTK-related signaling pathways represent a more effective approach for cancer treatment (Liu et al., 2020; Beljkas et al., 2023; Shirbhate et al., 2024). Several such compounds were developed with distinct target combinations. Compound 12L, a dual-target HDAC-mTOR inhibitor, induced G_0_/G_1_ arrest and apoptosis in tumor cells by increasing histone H3 acetylation and suppressing mTOR downstream effectors. Notably, in a multiple myeloma xenograft model, it markedly inhibited tumor growth with minimal toxicity (Chen et al., 2019). Extending this concept to the RAS signaling network, patent CN116444447B disclosed SH-4, a quinazoline hydroxamic acid targeting SOS1 and HDACs. This compound potently inhibited tumor cell proliferation and induced apoptosis, demonstrating strong *in vitro* and *in vivo* anticancer activity (Shukla et al., 2025). Compound 8t, a dual-target inhibitor of SHP2 and HDACs, showed superior anticancer efficacy *in vitro* and *in vivo* compared with SHP099 (an SHP2-selective inhibitor) and vorinostat (Liu M. et al., 2022).

### 6.2 Targeting HDACs for sensitization to proteasome inhibitors

Proteasome inhibitors are widely used in hematologic malignancies and selected solid tumors (Fricker, 2020). HDACis can enhance their anticancer activity through diverse mechanisms. Bortezomib, the first approved proteasome inhibitor for multiple myeloma (Zhang et al., 2020), exhibited synergistic effects with several HDACis. For instance, in head and neck cancer cells, the class IIa HDACi CHDI0039 enhanced bortezomib activity and may possess antitumor potential in platinum-resistant disease (Schrenk et al., 2023). Beyond broad-spectrum HDACis, selective targeting of HDAC6 also showed promise in overcoming bortezomib resistance. In bortezomib-resistant multiple myeloma cells, the HDAC6 inhibitor A452 suppressed ERK and NF-κB signaling; when combined with bortezomib or carfilzomib, it further inhibited NF-κB and STAT3 signaling, downregulated LMP2 and LMP7, and induced apoptosis (Lee et al., 2021). Similarly, ACY-1215 synergized with bortezomib by prolonging endoplasmic reticulum stress and promoting cell death (Santo et al., 2012). HDACis also synergize with other proteasome inhibitors, including carfilzomib, oprozomib, and ixazomib. In head and neck squamous cell carcinoma, vorinostat enhanced the effects of carfilzomib and oprozomib by promoting apoptotic signaling (Zang et al., 2014). Likewise, belinostat prevented compensatory proteasome recovery upon ixazomib treatment by suppressing *NFE2L2* and proteasome gene transcription, thereby promoting synergistic cytotoxicity in T-cell lymphoma and Hodgkin lymphoma cells (Passero et al., 2020). Overall, HDACis represent a promising strategy to sensitize tumor cells to proteasome inhibition in both hematologic malignancies and solid tumors.

### 6.3 Targeting HDACs for sensitization to other targeted therapies

HDACis can sensitize tumors to a broad range of targeted agents, including inhibitors of cell cycle regulators, DNA damage repair pathways, epigenetic regulators, anti-apoptotic proteins, and molecular chaperones.

#### 6.3.1 Cyclin-dependent kinase (CDK) inhibitors

Co-targeting HDACs and CDKs has shown synergistic antitumor activity. In acute myeloid leukemia, dinaciclib plus CAY10603 consistently suppressed clonogenic growth, induced caspase-dependent apoptosis, and downregulated key cell cycle regulators, including CDK2, CDK4/6 and their associated cyclins, while upregulating the expression of the CDK inhibitor p21 (Pottier et al., 2025).

#### 6.3.2 PARP inhibitors

HDACis also enhanced the efficacy of PARP inhibitors by impairing DNA repair and promoting apoptosis. Talazoparib plus romidepsin induced synergistic cell death in mycosis fungoides through suppressing the expression of DNA repair-related genes and activating the Blimp-1/BAX axis (Kruglov et al., 2020). In breast and ovarian cancer cells, HDACis combined with PARP inhibitors and decitabine showed synergistic antitumor effects by inhibiting PARylation, reducing c-Myc expression, and promoting DNA damage (Valdez et al., 2024).

#### 6.3.3 EZH2 and BET inhibitors

HDACis also synergized with inhibitors of other epigenetic regulators, such as EZH2 and BET proteins. In CRPC, EZH2 inhibitors plus vorinostat induced histone H3 demethylation/acetylation, restored EZH2 target gene expression, and promoted apoptosis (Schade et al., 2023). In germinal center-derived lymphomas, the combination of EZH2 inhibitors and romidepsin yielded synergistic efficacy by promoting RbAp46/48 acetylation and dissociating the PRC2 complex (Lue et al., 2019). BET inhibitors also cooperated with HDACis in pancreatic ductal adenocarcinoma and urothelial carcinoma by downregulating oncogenic drivers such as c-Myc, Survivin, and BCL-2 family members (Mazur et al., 2015; Hölscher et al., 2018).

#### 6.3.4 Dual-target inhibitors

Beyond conventional drug combinations, dual-target inhibitors incorporating HDAC inhibition have emerged as an attractive strategy (Smalley et al., 2020). In addition to dual-action inhibitors targeting HDACs and RTK-related signaling pathways discussed above, JAK/HDAC and HSP90/HDAC dual-target inhibitors showed superior antitumor activity with reduced toxicity compared with single agents in hematologic malignancies and aggressive solid tumors (Chu-Farseeva et al., 2018; Liang et al., 2019; Wu et al., 2021; Citarella et al., 2025).

In conclusion, HDACis enhance the efficacy of multiple targeted therapies, and HDAC-based dual-target inhibitors offer the potential to further improve antitumor activity with reduced toxicity. Further optimization of HDAC-based combination strategies will help expand their application across a broader spectrum of cancers.

## 7 Targeting HDACs for sensitization to endocrine therapy

Endocrine therapy exerts antitumor effects by disrupting hormone-mediated oncogenic signaling in hormone-driven malignancies (Will et al., 2023; Burciu et al., 2025). However, resistance to these agents remains a major clinical challenge. HDAC inhibition has emerged as a promising strategy to enhance the efficacy of endocrine-based therapies, particularly in breast and prostate cancers (Figure 6).

**FIGURE 6:**
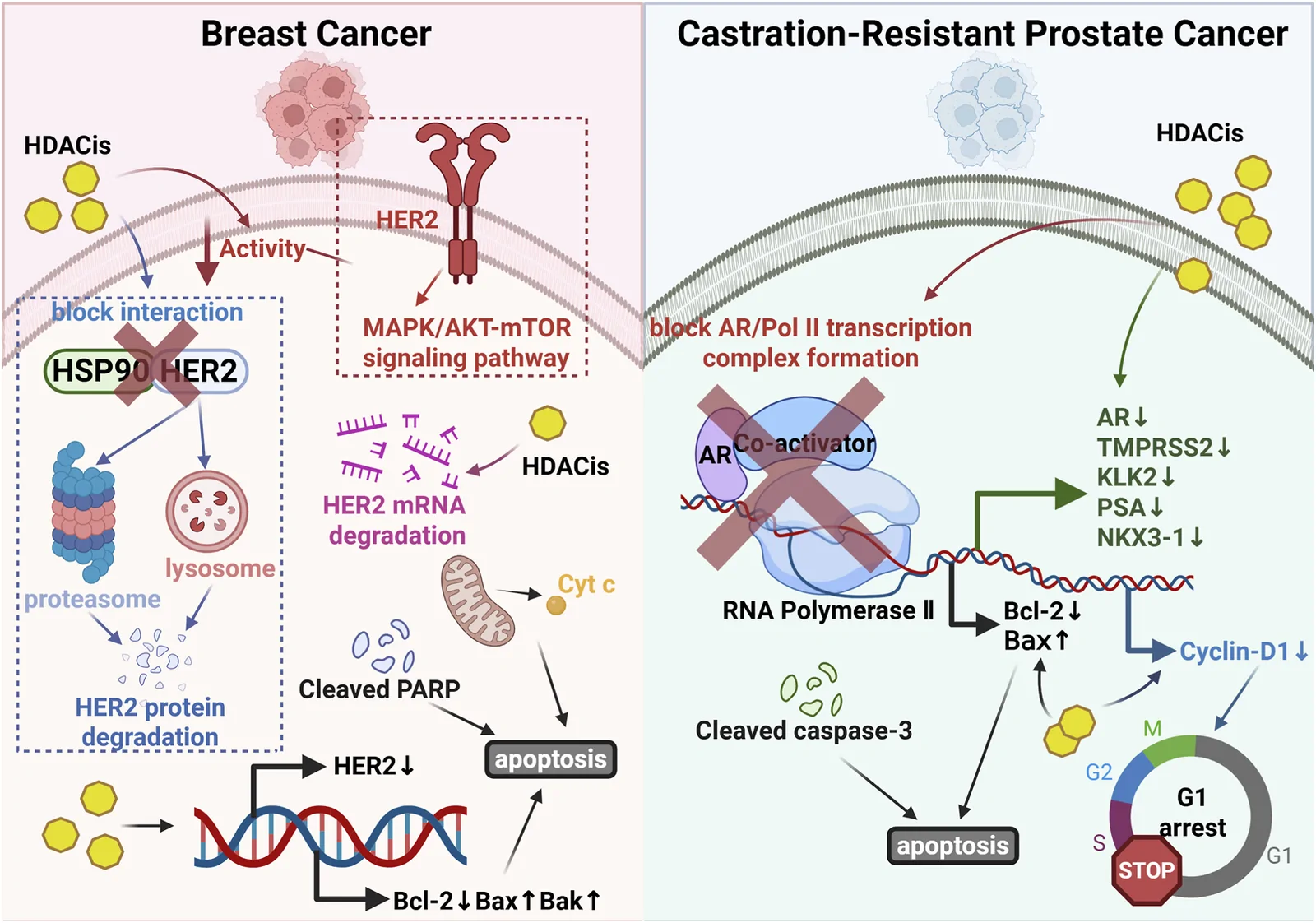
Targeting HDACs to sensitize tumors to Endocrine Therapy. HDACis enhance the efficacy of endocrine therapy in breast and prostate cancers by modulating key signaling pathways critical for hormone receptor function and cancer cell survival. Created with BioRender.com.

Preclinical studies have established a mechanistic rationale for combining HDACis with endocrine therapy in breast cancer. In tamoxifen-resistant ER-positive models, prolonged exposure to 4-hydroxytamoxifen led to increased ER expression, loss of PGR expression, and upregulation of BCL-2; HDAC inhibition restored tamoxifen sensitivity by reversing these aberrant transcriptional programs, reducing BCL-2 expression, and promoting growth arrest and apoptosis (Raha et al., 2015). In a separate study using ER-positive breast cancer cells, HDAC inhibition redirected the response to tamoxifen from growth arrest toward apoptosis, accompanied by downregulation of BCL-2 expression and upregulation of BAX and BAK expression, manifesting in cytochrome C release and PARP cleavage. (Thomas et al., 2011). In acquired letrozole-resistant models, entinostat re-sensitized tumors to aromatase inhibitors by suppressing HER2 signaling through dual mechanisms, disrupting the HSP90-HER2 interaction to promote HER2 protein degradation, and reducing HER2 mRNA levels and stability. These effects collectively suppressed MAPK/AKT-mTOR signaling and reversed letrozole resistance (Sabnis et al., 2013).

Consistent with these preclinical findings, clinical studies have provided further support for combining HDAC inhibition with endocrine therapy. In the phase 3 ACE trial, tucidinostat plus exemestane improved progression-free survival (PFS) compared with placebo plus exemestane in patients with HR^+^/HER2^−^ advanced breast cancer who had progressed after prior endocrine therapy, although grade 3–4 hematologic adverse events occurred more frequently with tucidinostat plus exemestane (Jiang et al., 2019). Similarly, a phase 3 trial demonstrated that entinostat combined with exemestane significantly prolonged PFS in patients with endocrine-resistant HR^+^ advanced breast cancer, with manageable toxicity (Xu et al., 2023). Supporting these results, a meta-analysis confirmed that adding HDACis to exemestane improved PFS compared with exemestane alone in HR^+^ advanced breast cancer patients (Xu et al., 2022). Moreover, in patients with HR^+^/HER2^−^ advanced breast cancer previously treated with CDK4/6 inhibitors, the combination of tucidinostat, metronomic capecitabine, and endocrine therapy demonstrated antitumor efficacy and was generally well tolerated. (Wu et al., 2026). Collectively, these findings suggest the potential of HDAC inhibition to enhance endocrine sensitivity in breast cancer.

Beyond breast cancer, HDACis can also enhance the efficacy of androgen receptor (AR)-targeted therapies in prostate cancer. In CRPC, HDAC1 and HDAC3 supported AR-mediated transcription. Thus, HDACis reduced the expression of AR target genes such as *TMPRSS2*, primarily by preventing the assembly of coactivator/RNA polymerase II complex after AR binding to target gene enhancers (Welsbie et al., 2009). Consistently, the HDAC inhibitor ZJH-1 induced G1-phase cell cycle arrest accompanied by Cyclin D1 downregulation and promoted intrinsic apoptosis, as evidenced by caspase-3 cleavage, BAX upregulation, and BCL-2 downregulation in CRPC cells (Bao et al., 2025). In addition, dual-function compounds with both antiandrogenic and HDAC-inhibitory properties showed promising anticancer activity (Gryder et al., 2013). For example, SBI-46 suppressed CRPC cell growth by downregulating both full-length AR and AR splice variants and by potently targeting HDAC isoforms 1 and 6 (Chandrasekaran et al., 2023).

In conclusion, HDACis can enhance the efficacy of endocrine therapy in both breast cancer and CRPC, particularly in the setting of acquired resistance. Future studies should focus on validating these findings in larger patient cohorts and exploring additional combination strategies to further optimize clinical outcomes.

## 8 Targeting HDACs for sensitization to immunotherapy

HDACis have shown promising potential to sensitize tumors to immunotherapy. They can enhance the efficacy of immune checkpoint blockade (ICB), improve CAR T- and CAR NK-cell therapy, and regulate multiple immune cell populations, including T cells, NK cells, macrophages, and MDSCs, thereby strengthening antitumor immune responses (Figure 7).

**FIGURE 7:**
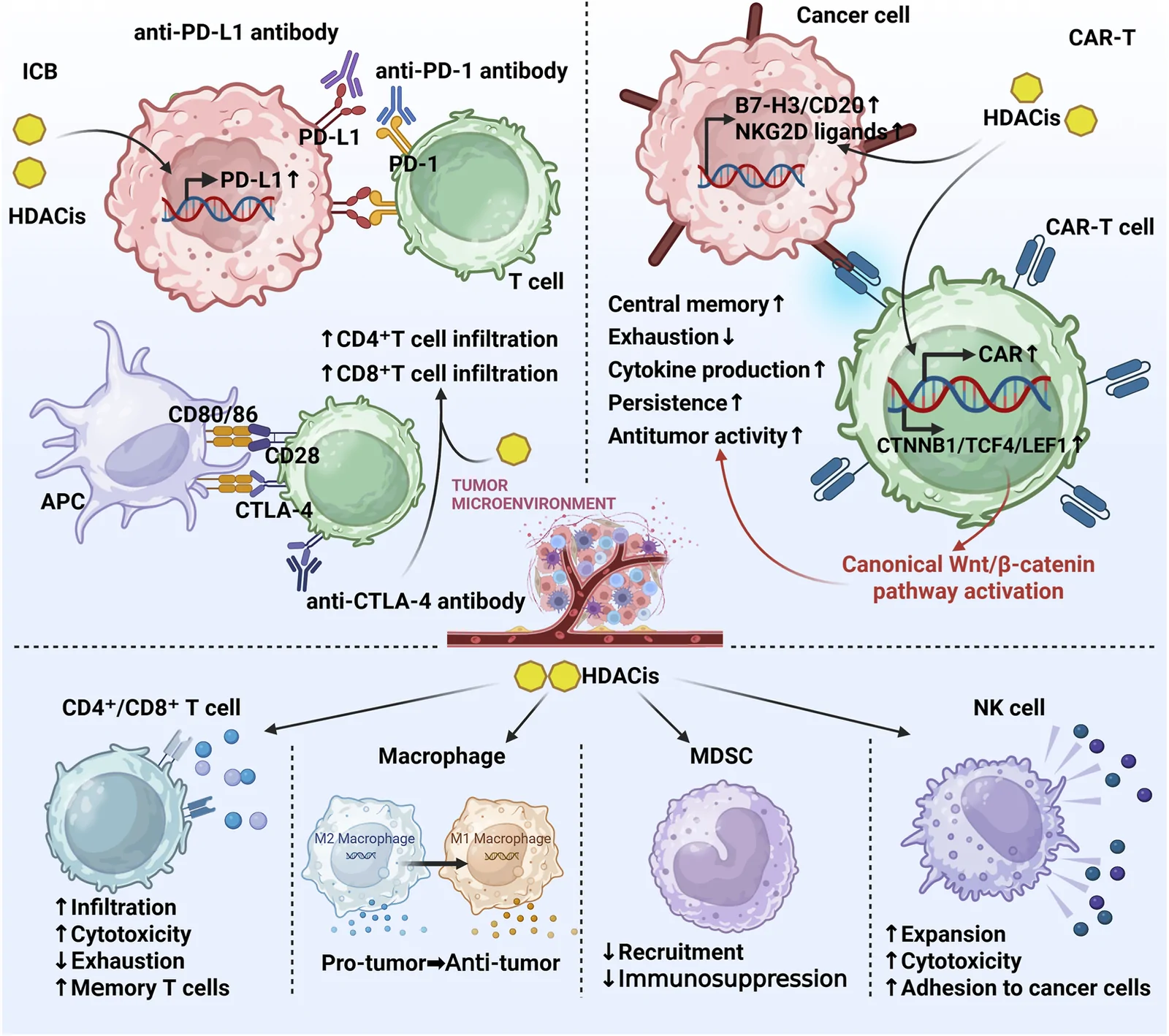
Targeting HDACs to sensitize tumors to Immunotherapy. HDACis synergize with ICB therapy, enhance CAR-T cell efficacy, augment T cell function and reverse exhaustion, boost NK cell cytotoxicity, reprogram macrophage phenotype, and attenuate MDSC-mediated immunosuppression, thereby potentiating antitumor immunity. Created with BioRender.com.

### 8.1 Targeting HDACs for sensitization to ICB therapy

Immune checkpoint blockade (ICB) reverses tumor immune evasion by inhibiting checkpoint pathways and restoring the tumor-killing capacity of immune effector cells (Morad et al., 2021; Aliazis et al., 2025). HDACis can further enhance tumor responsiveness to ICB therapy.

#### 8.1.1 Targeting HDACs for sensitization to PD-1/PD-L1 inhibitors

The PD-1/PD-L1 signaling axis represents a central immune checkpoint exploited by tumors to evade immune surveillance. By blocking this interaction, PD-1/PD-L1 inhibitors restore antitumor immune responses (Keir et al., 2008; Lee et al., 2024; Zhao et al., 2026). Increasing evidence suggests that HDACis can further enhance the anticancer efficacy of PD-1/PD-L1 blockade. Mechanistically, HDACis can modulate PD-L1 expression and the TME in a context-dependent manner. In melanoma, class I HDACis increased PD-L1 expression by enhancing histone acetylation around the *PD-L1* gene locus, thereby improving responsiveness to PD-1 blockade and delaying tumor progression in preclinical models (Woods et al., 2015). Similarly, in soft tissue sarcomas, chidamide upregulated PD-L1 and modulated the TME, exhibiting synergistic antitumor activity with PD-1 inhibitors in both animal models and patients with advanced disease (Que et al., 2021). In B-cell lymphoma, HDAC3 acted as a transcriptional repressor of the *PD-L1* gene. Treatment with pan-HDAC or selective HDAC3 inhibitors inhibited HDAC3, promoted BRD4 recruitment to the *PD-L1* promoter, and led to transcriptional activation, ultimately improving the response to anti-PD-L1 therapy (Deng et al., 2019). In anaplastic thyroid carcinoma, both vorinostat and VPA induced cell cycle arrest and increased PD-L1 expression, providing a rationale for combining these agents with ICB (Hegedűs et al., 2020). Beyond direct modulation of PD-L1, entinostat has also been shown to exert synergistic antitumor effects with PD-1 inhibitors, partly by suppressing MDSCs and alleviating the immunosuppressive TME (Orillion et al., 2017).

#### 8.1.2 Targeting HDACs for sensitization to CTLA-4 inhibitors

Cytotoxic T-lymphocyte-associated protein 4 (CTLA-4) is an inhibitory receptor expressed on activated T cells (Yang et al., 2021). It outcompetes CD28 for binding to B7-1 (CD80) and B7-2 (CD86) on antigen-presenting cells (APCs), delivering suppressive signals that limit CD4^+^/CD8^+^ T-cell proliferation. Tumors exploit this pathway to blunt antitumor immunity, and CTLA-4 inhibitors restore immune activation by preventing the interaction between CTLA-4 and B7 (Buchan, 2024; Cao et al., 2025). Fas-FasL-dependent activation-induced cell death (AICD) of T cells is proposed as a major contributor to impaired antitumor immunity (Yajima et al., 2019; Gadelkarim et al., 2026). Cao et al. showed that HDACis suppressed AICD of intratumoral CD4^+^ T cells by suppressing NFAT1-dependent FasL expression, thereby enhancing antitumor immunity and suppressing melanoma progression. Furthermore, combination treatment with HDACis and anti-CTLA-4 antibodies increased CD4^+^ T cell infiltration into tumors and produced synergistic antitumor effects (Cao et al., 2015). In immunoresistant breast and pancreatic cancers, entinostat promoted tumor immunogenicity, thereby sensitizing tumors to CTLA-4 blockade. In murine models, combining entinostat with CTLA-4 inhibitors improved tumor-free survival and increased intratumoral CD8^+^ T cell infiltration (Christmas et al., 2018).

### 8.2 Targeting HDACs for sensitization to CAR T-Cell therapy

CAR T cells are genetically engineered T cells that recognize tumor antigens through chimeric antigen receptors (CARs) and exert potent antitumor activity (Bot et al., 2026). Emerging evidence indicates that HDACis can further enhance the efficacy of CAR T-cell therapy. Mechanistically, HDACis improve CAR T-cell function through multiple distinct mechanisms. Class I HDACis (M344 and chidamide) reduced HDAC1 levels and increased H3K27 acetylation, leading to upregulation of *TCF4*, *LEF1* and *CTNNB1* and activation of canonical Wnt/β-catenin signaling in CAR T cells. This signaling cascade promoted the maintenance of memory-like phenotypes and limited exhaustion, thereby supporting the sustained antitumor activity of CAR T cells (Zhu et al., 2024). In addition to modulating CAR T-cell intrinsic properties, HDACis also enhanced CAR T-cell efficacy by increasing target antigen expression on tumor cells. In B-cell malignancies, HDACis increased H3K9 acetylation at the *CD20* promoter, upregulated *CD20* expression and enhanced the cytotoxicity of CD20-directed CAR T cells (Xu et al., 2019). In acute myeloid leukaemia and T-cell acute lymphoblastic leukaemia, HDACis induced NKG2D ligand expression on tumor cells, thereby improving the killing capacity of NKG2D-targeting CAR T cells (Driouk et al., 2020). In solid tumors, vorinostat increased B7-H3 expression on tumor cells, promoted the surface expression of B7-H3-specific CARs on CAR T cells, and suppressed immunosuppressive molecules such as CTLA-4, collectively enhancing the efficacy of B7-H3-specific CAR-T therapy (Lei et al., 2021). In addition, combining celecoxib with HDACis was shown to partially overcome acquired resistance to CD19-targeting CAR T cells in non-Hodgkin lymphoma by modulating apoptotic signaling pathways (Torres-Collado and Jazirehi, 2018).

### 8.3 Modulatory effects of HDACis on the antitumor efficacy of diverse immune cells

#### 8.3.1 Modulatory effects of HDACis on T Cells

Effective T cell infiltration into the TME is critical for antitumor immunity, and emerging evidence indicates that HDACis can actively facilitate this process. For instance, in soft tissue sarcomas, chidamide has been shown to directly promote CD8^+^ T cell infiltration, and its combination with PD-1 blockade led to marked tumor regression (Que et al., 2021). Similarly, in melanoma, combining HDACis with anti-CTLA-4 antibodies synergistically enhanced CD4^+^ T cell infiltration into tumors, thereby potentiating antitumor activity (Cao et al., 2015).

Beyond infiltration, HDACis also enhance the cytotoxic function of CD8^+^ T cells through epigenetic reprogramming. Mechanistically, Wang et al. demonstrated that chidamide enhanced chromatin accessibility at the promoters of genes encoding effector molecules in CD8^+^ T cells, resulting in their upregulated expression and augmented antitumor capabilities (Wang et al., 2025). Moreover, HDAC1/2-mediated repression of ALOX15B contributed to an immunosuppressive TME and impaired CD8^+^ T cell cytotoxicity; importantly, chidamide restored ALOX15B expression, reactivated the TAP1/MHC-I pathway, and enhanced tumor cell apoptosis (Wang et al., 2026).

The persistence of T cells is essential for sustaining long-term antitumor responses, particularly in the context of adoptive cell therapy, where resistance to exhaustion and long-term survival are key determinants of success. Central memory CD8^+^ T cells, characterized by their superior self-renewal capacity, are therefore considered favorable products for adoptive cell therapy (Klebanoff et al., 2006; Li et al., 2025). In this regard, HDACis show promise in promoting T cell persistence. Wang et al. reported that HDACis combined with IL-21 could reprogram differentiated human CD8^+^ T cells into central memory-like T cells, accompanied by increased expression of CD28 and CD62L (Wang J. et al., 2020). In addition, selective HDAC6 inhibitors such as ACY-1215 and ACY-241 have been shown to enhance T cell persistence by reducing exhaustion, promoting the expansion of central memory T cells, and attenuating regulatory T cell (Treg)-mediated immunosuppression, thereby supporting durable antitumor responses in metastatic melanoma (Laino et al., 2019).

#### 8.3.2 Regulatory effects of HDACis on natural killer (NK) cells

NK cells are key effector cells of innate immunity that directly eliminate tumor cells and remodel the TME by secreting IFN-γ, TNF-α, and various chemokines (Page et al., 2024). Emerging evidence indicates that HDACis regulate NK cell function through multiple complementary mechanisms, ranging from enhancing proliferation and activating signaling pathways to improving target cell recognition and conjugation. At the level of NK cell expansion and activation, low-dose dacinostat and panobinostat have been shown enhance *IL-2*-driven proliferation of NK92 cells, primary NK cells, and CD19-CAR-NK92 cells without impairing their effector functions (Zheng J. et al., 2023). Mechanistically, dacinostat activated the JAK-STAT signaling pathway and promoted cell cycle progression in these cells (Zheng J. et al., 2023). Beyond proliferation, HDACis also facilitate the physical interaction between NK cells and tumor targets. For instance, panobinostat increased the expression of genes involved in cell adhesion and tight junction formation, thereby promoting conjugate formation between NK cells and tumor cells. Furthermore, it modulated the expression of activating receptors on NK cells and their corresponding ligands on tumor cells, ultimately enhancing tumor cell cytolysis (Afolabi et al., 2021). A key axis targeted by multiple HDACis is the NKG2D pathway. Entinostat and VPA upregulated NKG2D expression on NK cells and NKG2D ligands on tumor cells, thereby strengthening NK cell-mediated cytotoxicity in colon cancer and acute myeloid leukemia (Diermayr et al., 2008; Zhu et al., 2015). Consistently, romidepsin significantly induced the expression of the NKG2D ligands MICA/B in both rituximab-sensitive and rituximab-resistant Burkitt lymphoma cells, resulting in enhanced NK cell cytotoxicity (Chu et al., 2017). In a related application of adoptive cell therapy, HDACis may improve CAR NK-cell therapy by preserving CAR expression during *ex vivo* expansion, thereby enhancing CAR NK cell cytotoxicity against CD138-positive multiple myeloma cells (Jo et al., 2025).

#### 8.3.3 Regulatory effects of HDACis on macrophages

Macrophages are key innate immune cells within the TME. Classically activated M1-like macrophages mediate antitumor immunity, whereas alternatively activated M2-like macrophages promote tumor progression and immune evasion (Zhang et al., 2024; Guan et al., 2025). Accumulating evidence indicates that HDACis can enhance antitumor immunity by modulating macrophage polarization and effector functions, primarily by shifting the balance from M2 toward an M1-like phenotype. Low-dose TSA promoted an antitumor macrophage phenotype and enhanced antitumor immune responses. Mechanistically, it significantly decreased the expression of M2-associated markers such as *Arg1*, *Cd206*, and *Fizz1*, while increasing the expression of M1-associated markers such as *Nos2* and *Il-6* (Li et al., 2021). Beyond broad-spectrum HDACis, selective targeting of specific HDAC isoforms also showed efficacy. *HDAC2* overexpression in M2-like macrophages was associated with unfavorable outcomes in patients with lung cancer; conversely, HDAC2 inhibition promoted a shift toward an M1-like phenotype, modulated cytokine signaling, and suppressed tumor cell proliferation, migration, and angiogenesis (Zheng X. et al., 2023). Similarly, HDAC6 contributed to tumor progression by promoting M2-like macrophage polarization, whereas HDAC6 inhibition increased M1-like macrophage recruitment and reduced tumor growth (Jiang et al., 2025). In addition to modulating polarization, HDACis also enhanced macrophage-mediated phagocytosis. In *MYC*-driven medulloblastoma, the class I HDAC inhibitor tacedinaline induced NF-κB-TGM2-dependent tumor inflammation and increased the cell-surface expression of engulfment signals, such as calreticulin, thereby enhancing macrophage-mediated phagocytosis following anti-CD47 treatment (Marquardt et al., 2023).

#### 8.3.4 Regulatory effects of HDACis on myeloid-derived suppressor cells (MDSCs)

MDSCs are a heterogeneous population of immune cells that potently suppress the antitumor activity of CD4^+^ and CD8^+^ T cells. Their aberrant recruitment and activation critically contribute to an immunosuppressive TME (Kumar et al., 2016; Veglia et al., 2021). Low-dose TSA has been shown to inhibit the recruitment of MDSCs, thereby reducing their immunosuppressive effects and augmenting the anticancer immune response (Li et al., 2021). In murine models of lung cancer and renal cancer, entinostat reduced the expression of key immunosuppressive mediators including Arg-1, iNOS, and COX-2 in MDSCs. This effectively counteracted the suppressive activity of both granulocytic MDSCs (G-MDSCs) and monocytic MDSCs (M-MDSCs). Notably, when combined with PD-1 inhibitors, entinostat markedly improved antitumor efficacy (Orillion et al., 2017).

Nevertheless, the immune-cell effects described above should not be interpreted as universally beneficial, because HDAC inhibition can also impair immune-cell proliferation or effector function under particular treatment conditions.

### 8.4 Context-dependent immunomodulatory effects of HDACis

Although HDACis are increasingly investigated as immunotherapy sensitizers, their effects on immune cells are not uniformly stimulatory. Instead, these effects depend strongly on HDAC isoform selectivity, drug concentration, exposure duration, and treatment sequence. For example, in the presence of IL-2, the pan-HDAC inhibitors dacinostat enhanced the proliferation of NK92 cells without impairing their effector function only at low nanomolar doses. In contrast, treatment with dacinostat at concentrations above 500 nM markedly increased NK92 cell apoptosis, highlighting the dose-dependent nature of the immunological effects of these agents (Zheng J. et al., 2023). Exposure duration is also critical. Short-term treatment with bocodepsin (OKI-179) rapidly increased histone acetylation and the frequencies of CD4^+^ and CD8^+^ T cells in the blood, whereas prolonged exposure sustained excessive acetylation and suppressed cytokine production. Consistent with these findings, intermittent discontinuation of OKI-179 during anti-PD-1 therapy resulted in superior responses compared with continuous dosing (Holay et al., 2023). The sequence of administration may likewise influence therapeutic efficacy. Pretreatment with the selective HDAC6 inhibitor Nexturastat A for 10 days before anti-PD-1 therapy more effectively remodeled the tumor microenvironment than simultaneous combination treatment, particularly by increasing the M1/M2 macrophage ratio, thereby improving tumor control (Knox et al., 2019).

In addition, the immunological consequences of HDAC inhibition may vary substantially according to the targeted HDAC isoform. The class I-selective HDAC inhibitor entinostat has been shown to increase NKG2D expression and chromatin accessibility, thereby promoting IFIT1 transcription and activating the downstream STING-STAT4-IRF1 signaling axis. These effects enhance NK cell cytotoxicity and IFN-γ production (Idso et al., 2020). In contrast, other HDACis may impair NK cell activity. TSA (pan-HDACi), VPA (class I + IIa selective), and sodium butyrate (NaB) (class I + IIa selective) inhibited IFN-γ production by both CD56 (dim) and CD56 (bright) NK cells and reduced NK cell-mediated cytotoxicity against K562 target cells. Moreover, NK cells from normal mice treated *in vivo* with TSA displayed a diminished expression of NK1.1, NKG2D, and NKp46 and secreted reduced amounts of IFN-γ upon *ex vivo* stimulation with cytokines (Rossi et al., 2012).

Similar differences have been observed in the regulation of T cells. Entinostat transcriptionally downregulated Foxp3 and impaired the suppressive function of regulatory T cells (Tregs) without substantially affecting effector T cells (Shen et al., 2012). Conversely, treatment with the pan-HDAC inhibitor TSA increased the number of peripheral Tregs, promoted acetylation of Foxp3 and histones in Tregs, upregulated functionally relevant Treg-associated markers, including Foxp3, GITR, and CTLA-4, and increased Treg suppressive capability (Tao et al., 2007).

### 8.5 Clinical applications and challenges of targeting HDACs to augment the efficacy of immunotherapy

Clinical studies have demonstrated the potential of HDACi-based combination immunotherapies, although their efficacy and tolerability vary according to tumor type, HDAC isoform selectivity, dosing schedule, and combination regimen. In the phase 2 CAPability-01 trial, sintilimab, chidamide, and bevacizumab showed promising efficacy in patients with MSS/pMMR advanced colorectal cancer. The triplet regimen improved PFS and objective response rates compared with sintilimab plus chidamide alone, but was associated with a higher incidence of adverse events (Wang et al., 2024). In another phase II trial, pembrolizumab plus vorinostat showed moderately promising antitumor activity in recurrent or metastatic head and neck squamous cell carcinoma, but the response rate was relatively low in salivary gland carcinoma, and toxicity was higher than previously reported with pembrolizumab monotherapy (Rodriguez et al., 2020). However, not every combination strategy translated into clinical benefit. Bevacizumab plus vorinostat failed to improve outcomes in recurrent glioblastoma, and panobinostat combined with rituximab failed to increase response rates in recurrent diffuse large B-cell lymphoma (Assouline et al., 2016; Ghiaseddin et al., 2018).

Collectively, these findings underscore that, although HDACis can potentiate immunotherapy through diverse mechanisms, their successful clinical translation will require careful patient selection, biomarker-driven trial design, rigorous toxicity assessment, and validation of optimized combination strategies in larger patient cohorts. Future clinical trials should also systematically optimize the dose, exposure duration, and treatment sequence to maximize therapeutic benefit while minimizing unintended suppression of antitumor immunity.

## 9 Discussion

Considerable enthusiasm surrounds the use of HDACis in combination with other anticancer drugs. However, the mechanisms responsible for HDACi-mediated sensitization are still not fully understood, and clinical trial data have shown variability (Table 2). Addressing these gaps should therefore be a priority in future research (Figure 8).

**FIGURE 8:**
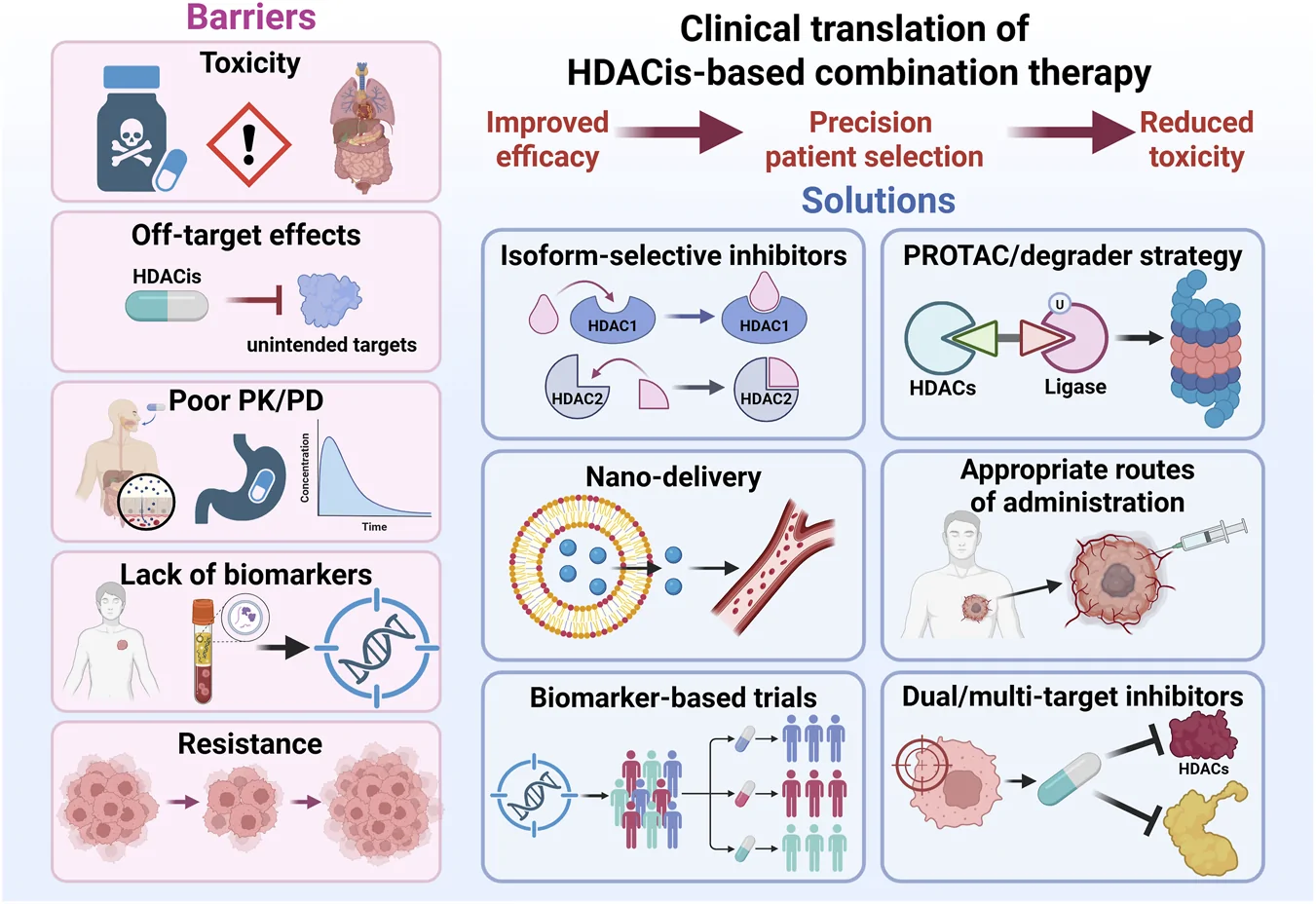
Barriers to clinical translation of HDACis-based combination therapy and solutions. Clinical development of HDACis-based combination strategies is limited by toxicity, off-target effects, poor PK/PD properties, lack of predictive biomarkers, and therapeutic resistance. Emerging solutions include isoform-selective inhibitors, PROTACs and other degrader technologies, nanoparticle delivery systems, optimized administration routes, biomarker-based trials, and dual-/multi-target inhibitors. These advances may enable safer, more precise, and more effective HDACis-based cancer therapies. Created with BioRender.com.

The off-target effects and toxicity of HDACis pose significant challenges to their clinical application. There is an urgent need to develop isoform-selective HDAC inhibitors that target specific HDAC isoforms in defined cancer types. To this end, techniques such as single-cell sequencing and spatial transcriptomics should be employed to elucidate the expression profile of critical HDAC subtypes not only in tumor cells but also in other cell types within the TME, such as CAF and TAM, across diverse tumor types. Informed by these findings, highly selective HDACis can be designed to target specific HDAC isoforms in tumor cells, thus improving treatment accuracy while minimizing damage to healthy cells. An alternative strategy involves nanomedicine and targeted delivery techniques to develop highly effective, low-toxicity drug delivery systems. For example, the TME-responsive nanocarriers can facilitate the targeted release of HDACis within tumor tissues. Similarly, local delivery techniques, such as intratumoral injection or sustained-release formulations, may be utilized to mitigate drug toxicity.

Moreover, since HDACis augment the efficacy of anticancer agents and produce synergistic antitumor effects, the development of HDAC-based dual-target or multi-target inhibitors represents a promising direction. For instance, dual-target inhibitors can be designed to co-target HDAC with RAS, PARP, or VEGFR. Similarly, triple-target inhibitors that combine targeted therapy, epigenetic modulation, and immunotherapy may achieve a threefold effect comprising cellular killing, enhanced drug sensitivity, and immune evasion prevention. By attaining synergistic inhibition of multiple pathways with a single compound, these inhibitors can consequently diminish the overall toxicity and minimize drug-drug interactions.

Ultimately, biomarkers should be assessed to determine a patient’s suitability for a given HDACi-based combination regimen. For example, for HDACi-immunotherapy regimens, biomarkers associated with efficacy, including T cell infiltration features, MHC molecule expression levels, and HDAC subtype expression profiles, can be evaluated. These markers can then be used to establish a patient eligibility scoring system, ensuring appropriate patient stratification and avoiding the use of ineffective treatments.

## 10 Conclusion

As an important class of epigenetic agents, the significance of HDACis in cancer therapy extends far beyond monotherapy. HDACis exhibit potent synergistic efficacy when combined with various therapeutic modalities, including chemotherapy, radiotherapy, targeted therapy, endocrine therapy, and immunotherapy. By modulating key cellular processes such as chromatin relaxation, impairment of DNA damage repair, induction of apoptosis, inhibition of cell cycle progression, inhibition of oncogenic signaling pathways, and remodeling of the immune microenvironment, HDACis offer a promising strategy for enhancing the efficacy of the aforementioned therapies in preclinical studies. Clinical trials have also provided preliminary evidence of the safety and efficacy of these combination regimens. Nevertheless, drug toxicity, off-target effects, and acquired resistance continue to limit the long-term effectiveness of HDACi-based therapies, while limited clinical translation remains a major challenge. As research progresses, we are optimistic that these obstacles will be overcome, and further investigation of HDACis and their combination with other therapies will ultimately transform clinical cancer therapy.
